# MIND: multimodal integration with neighbourhood-aware distributions

**DOI:** 10.1038/s41467-026-74413-1

**Published:** 2026-06-24

**Authors:** Hanwen Xing, Christopher Yau

**Affiliations:** 1 Nuffield Department for Women’s and Reproductive Health, University of Oxford, Oxford, UK; 2 Health Data Research UK, London, UK

**Keywords:** Machine learning, Data integration, Software

## Abstract

Multimodal data integration combines different data modalities to improve predictive and classification performance. In biology, multi-omics profiling has become a powerful tool for applications such as cancer patient stratification. However, integration of multi-omics data remains challenging because of missingness and inherent heterogeneity. Methods such as imputation and sample exclusion often rely on strong assumptions that could lead to information loss or distortion. To address these limitations, we propose MIND (Multimodal Integration with Neighbourhood-aware Distributions), which learns patient-specific embeddings from incomplete multi-omics data using a multimodal Variational Autoencoder with a data-driven prior. We inject neighbourhood structure of the observed dataset, encoded as affinity matrices, into the prior, penalising latent configurations when neighbourhood structures in data and latent spaces diverge. MIND handles high missing rates, unbalanced missingness patterns, and low signal-to-noise ratios robustly. Compared with existing integration methods, MIND achieves better performance on downstream tasks on both synthetic and real data.

## Abstract (web summary)

Multi-omics data integration is challenging due to missingness and inherent heterogeneity, current methods rely on assumptions that can cause information distortion. Here, the authors propose MIND, a platform that learns patient-specific embeddings from incomplete datasets.

## Introduction

Multi-omics data integration has become essential for understanding complex biological systems and advancing precision medicine^1–3^. Modern high-throughput technologies enable simultaneous profiling of genomics, transcriptomics, proteomics, and epigenomics from the same samples, each providing complementary views of cellular function^4–6^. However, integrating these heterogeneous, high-dimensional datasets presents significant computational challenges.

**Fig. 1:**
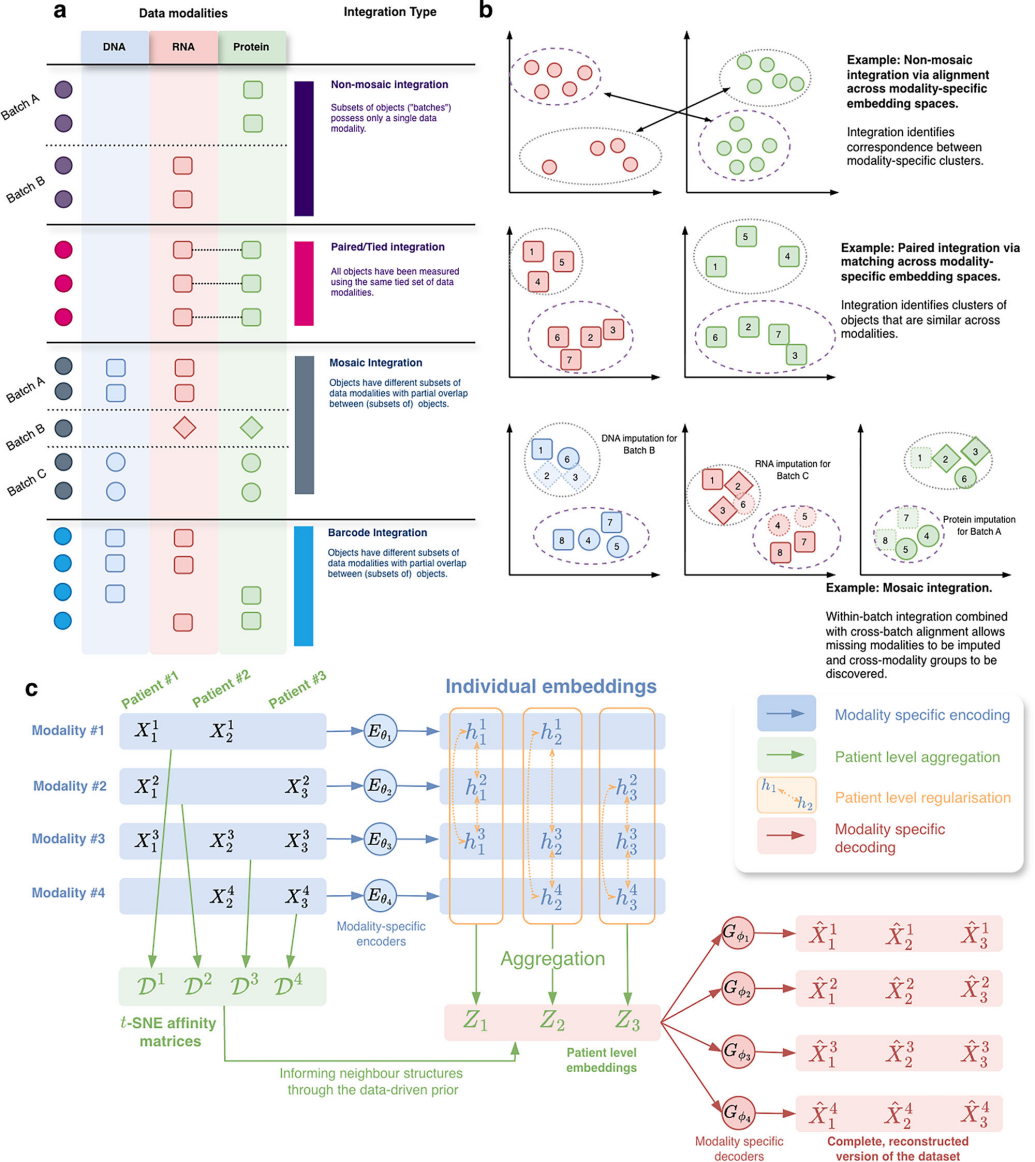
Barcode integration with MIND. **a** Illustration of different integration problem types. Mosaic integration arises when missingness is structured at the batch level: all objects within a batch share the same subset of available modalities, so that only a small number of distinct missingness patterns exist across the cohort. In contrast, barcode integration arises when missingness varies at the individual level. **b** The corresponding solutions. **c** Schematic illustration of MIND using an incomplete multiomics dataset with *M* = 4 modalities and *N* = 3 patients. Available ${X}_{n}^{m}$ s in the incomplete dataset are first mapped to individual embeddings ${h}_{n}^{m}\,{{\rm{s}}}$ by modality-specific encoders ${\{{E}_{{\theta }_{m}}\}}_{m=1}^{M}$. ${h}_{n}^{m}\,{{\rm{s}}}$ associated with the same patient *n* are then aggregated into a patient level embedding *Z*_*n*_ with a cross-modal regularisation term (Eq (4)) that penalises discrepancies between ${h}_{n}^{m}\,{{\rm{s}}}$ from different modalities *m* = 1, . . . , *M* of the same patient *n*, encouraging modality-specific encoders to produce similar representations. Each ${X}_{n}^{m}$ is reconstructed or predicted by passing *Z*_*n*_ to the modality-specific decoder ${G}_{{\phi }_{m}}$. For each modality, a *t*-SNE affinity matrix ${{{\mathcal{D}}}}^{m}$ encoding neighbour structure of the relevant patients is computed. These matrices guide the neighbour structure of *Z*_*n*_s through the prior on them.

The nature of the form of data integration required depends on the experimental set up (Fig. 1a). One form of integration involves combining information from across batches of objects, where each batch is associated with a different data modality. For example, one may measure gene expression in some cells but only protein abundance in another set. Alignment approaches offered within single cell analysis packages such as Seurat^7^ or specific methods such as LIGER^8^ seek to align different data modalities to a common latent coordinate space where the correspondence between modality-specific groupings and patterns can be ascertained (Fig. 1b).

When all objects are measured using the same pair (or more) of tied modalities, shared groupings and patterns between objects can be examined directly across the different modalities. However, if some batches of objects only contain some modalities, while others contain a differing set of modalities, this gives rise to a “mosaic integration" problem. For example, in single-cell studies, the modality availability is almost entirely tied to batch identity and sequencing technology. Single cell multi-omics integration methods, such as MIDAS^9^, have been developed to address the mosaic integration problem by leveraging shared information across batch to implicit impute missing modalities using variational autoencoders. Alternatives such as scMoMaT^10^ adopt a matrix tri-factorisation framework to simultaneously learn cell and feature embeddings while StabMap^11^ uses shared features in each pair of the multi-omics datasets to learn the dependency of different datasets, and use it to projects all cells onto a common reference coordinate system. See Supplementary Table 1 for a comparison of the features of different methods.

Although mosaic integration naturally arises in single-cell data collected in batches, existing methods for single-cell mosaic integration cannot be directly applied to problems with more complex missingness patterns. In particular, when learning patient-level embeddings from incomplete multimodal cancer atlases such as TCGA^12^, patients do not admit a natural notion of batch, and each patient may be observed in an arbitrary subset of modalities. Consequently, modality missingness is not structured at the batch level, but instead varies across patients in a heterogeneous and nearly combinatorial manner. In this regime, the missingness pattern for each modality resembles a random “barcode" rather than a structured mosaic shared across samples. As a result, the key assumptions underlying single-cell mosaic integration methods - namely repeated modality combinations and batch-level overlap - are violated, rendering these methods inapplicable without substantial modification. We summarise these distinctions in Fig. 1a, b.

One approach to resolve this “barcode integration" problem is to use labelled data and adopting a supervised learning approach. MOGONET^13^ uses a Graph neural net (GNN)-based supervised learning framework which uses labels to aid in the alignment of modality-specific latent spaces. IntegrAO^14^ removes the labelling requirements and solves the barcode problem by fusing partially overlapping patient graphs across modalities and using GNNs to extract and align unified patient embeddings, thus enabling integration and classification in the presence of highly heterogeneous and arbitrary modality missingness. While DLVPM^15^ learns a shared latent representation across patients and modelling structured relationships between data modalities without requiring repeated modality overlap or batch structure, thus naturally accommodating arbitrary, combinatorial missingness patterns. However, DLVPM does not produce sample level embeddings for downstreaming tasks, and focuses primarily on capturing inter-modal dependencies.

Our work most resembles latent variable models such as MOFA^16^, MOVE^17^, and JASMINE^18^ which address the barcode problem by learning shared sample-level latent representations and marginalising over arbitrarily missing modalities, thereby avoiding the need for repeated modality overlap or batch-structured integration. MOFA achieves this with a linear factor model, while MOVE and JASMINE extend the framework using nonlinear VAE architectures with increasing flexibility.

In this work, we present Multimodal Integration with Neighbourhood-aware Distributions (MIND), a lightweight and conceptually general multimodal Variational Autoencoder (VAE) framework. Unlike existing methods that either rely on modality-specific heuristics or complex interaction architectures, MIND directly incorporates data-driven neighbourhood priors into the latent space. This design principle enables the model to maintain interpretable and biologically meaningful representations in biomedical domains.

MIND utilises *t*-SNE-derived affinity matrices to construct a data-driven prior that preserves neighbourhood structures from individual omics modalities, ensuring biologically meaningful patient-level representations while maintaining VAE’s probabilistic modelling framework. Additionally, MIND employs cross-modal regularisation to encourage consistency and information sharing between modality-specific encodings from the same patient, thereby stabilising the aggregation step while maintaining flexibility for arbitrary missing patterns.

When applied to multi-omics, we demonstrate MIND’s superior performance across synthetic datasets and real-world applications including The Cancer Genome Atlas^19^, the Childhood Cancer Multi-omics Atlas^20^, and the Cancer Cell Line Encyclopaedia^21^. Our method consistently outperforms or achieves performance on par with existing approaches such as JASMINE^18^, IntegrAO^14^, and MOVE^17^ in survival prediction, cancer classification, clustering, and data reconstruction tasks, providing a robust framework for multi-omics integration in diverse biological contexts.

## Results

### Methods overview

Our proposed method uses a multimodal VAE architecture to extract patient-level embeddings from incomplete multiomics datasets (note we use the term “patient” for ease of description here, but this could be a cell line or other object as appropriate for any specific application). These patient-level embeddings are learnt by first mapping all entries in the incomplete dataset to individual embeddings using a collection of modality-specific encoders, and then aggregating available individual embeddings associated with the same patient into a single embedding. Each of the patient-level embeddings is then passed to each of the modality-specific decoders to reconstruct the multiomics observed data, or predict the missing ones. A schematic illustration of the modelling architecture can be found in Fig. 1c. To align the learnt patient-level embeddings with the intrinsic clustering structure present in biological data, we put a data-driven prior that preserves neighbourhood structures from individual modalities using *t*-SNE-derived affinity matrices on the patient-level embeddings. This prior distribution penalises the configuration of the latent embeddings based on the discrepancy between patients’ neighbourhood structures in the data spaces and the latent space, and favours latent embeddings whose clustering structure resembles a consensus of clustering structures present in individual modalities. Additionally, a cross-modality regularisation that encourages consistency and information-sharing between individual embeddings of the same patient is used to capture inter-modality dependencies of the incomplete multiomics datasets. Implementation details of MIND can be found in Methods.

### Synthetic data

**Fig. 2:**
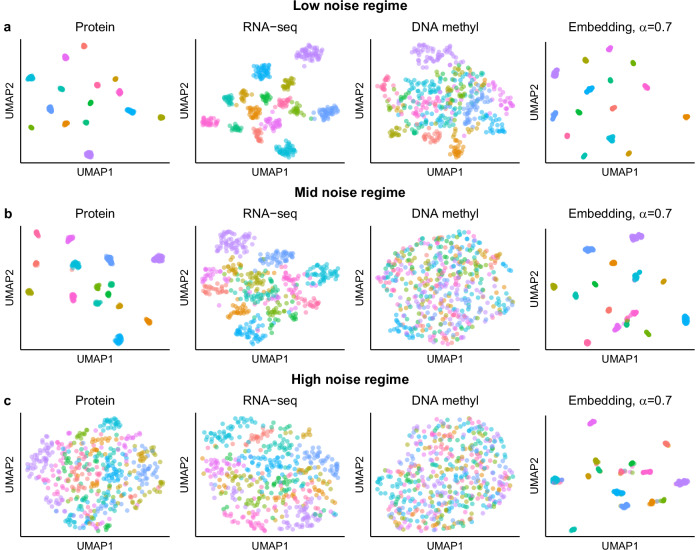
Visualisation of synthetic datasets and embeddings. **a** Low noise regime. Left to right: UMAP visualisation of the three modalities: Protein, RNA-seq, and DNA methylation. UMAP visualisation of the embeddings estimated by the proposed method at overlap ratio *α* = 0.7. **b** Similar figures under the mid noise regime. **c** Similar figures under the high noise regime. Source data for this figure is available in the Source Data file.

We first demonstrate the efficacy of the proposed method using synthetic multi-omics data generated by the InterSim CRAN package^22^ (Fig. 2). The synthetic data set consists of three modalities: DNA methylation, mRNA expression, and protein expression. Similarly to ref. ^14^, we set the number of patients *N* = 500 and the number of groups *C* = 15. To demonstrate the robustness of the proposed method, we consider three scenarios in which (1) all modalities show clear and consistent clustering patterns (2) some modalities are more noisy and less informative and (3) none of the modalities show clear clustering patterns. We refer to the three scenarios as the low, mid and high noise regime, and the corresponding synthetic data sets are generated by passing different parameters to InterSim (see Methods).

To mimic missingness patterns in real data, we follow^14^ and mask subsets of data from each of the modalities as follows: Given an overlap ratio *α*, we first randomly select ⌊*αN*⌋ patients as common samples (i.e., keep their data intact), then evenly distribute the remaining ⌈(1 − *α*)*N*⌉ patients among the three modalities as unique samples. For example, when *α* = 0.7 and *N* = 500, each of the three modalities would have 50 unique patients, and the remaining 350 patients are present in all three modalities. We tested our proposed method at overlap ratios *α* ∈ {0.1, 0.2, …, 0.9} in low, mid, and high noise regimes.

We compare our proposed method with three current state-of-the-art models: the VAE-based JASMINE^18^ and MOVE^17^, and the network-based IntegrAO^14^. We also include MSNE^23^ as a further baseline. We set the dimension of embeddings *d*_*l*_ = 64 for all methods. We apply these methods to the three synthetic datasets and compare the quality of the generated embeddings by investigating their performance on supervised and unsupervised downstream tasks.

**Fig. 3:**
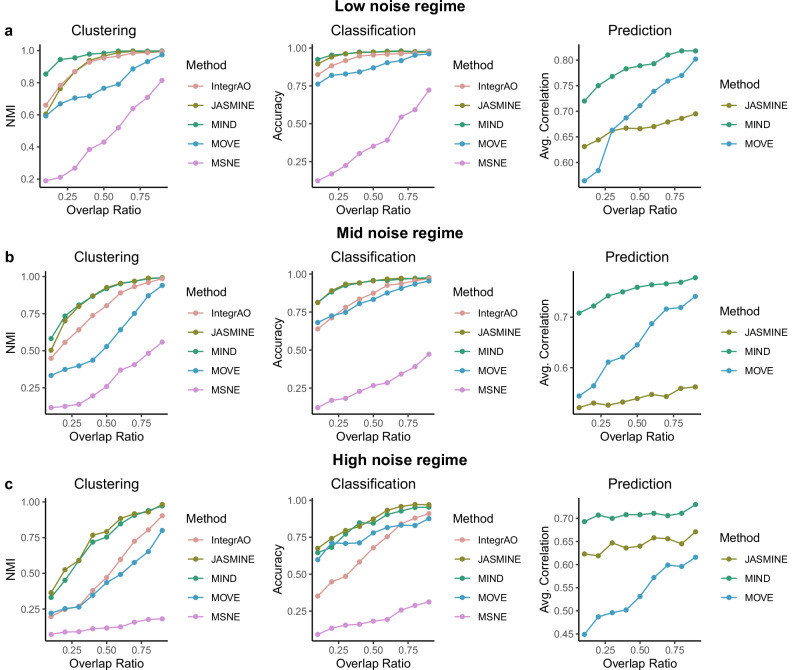
Performance on the three downstream tasks. **a** Results from the low noise datasets. Left to right: Normalised Mutual Information vs overlapping data ratio of different methods. Classification accuracy estimated by 10-fold CV of different methods. Prediction accuracy measured by the Pearson correlations between the masked portion of the synthetic data and the predicted, averaged over all modalities. **b** Similar results from the mid noise datasets. **c** Similar results from the high noise datasets. Source data for this figure is available in the Source Data file.

We first investigated the clustering performance of different methods. For each method, we apply spectral clustering with the true number of clusters *C* = 15 to the generated embeddings and compare their clustering results with the true group membership using normalised mutual information (NMI) as a metric. MIND exhibits higher or comparable NMI at all overlap ratios considered compared to other methods (Fig. 3).

We then considered two supervised tasks, classification and prediction. For classification, we train classifiers that predict patient groups from embeddings generated by each method using XGBoost^24^, and report 10-fold CV accuracies at different overlap ratios (Fig. 3). MIND outperformed IntegrAO, MOVE and MSNE in classification accuracy and had similar accuracy to JASMINE.

**Fig. 4:**
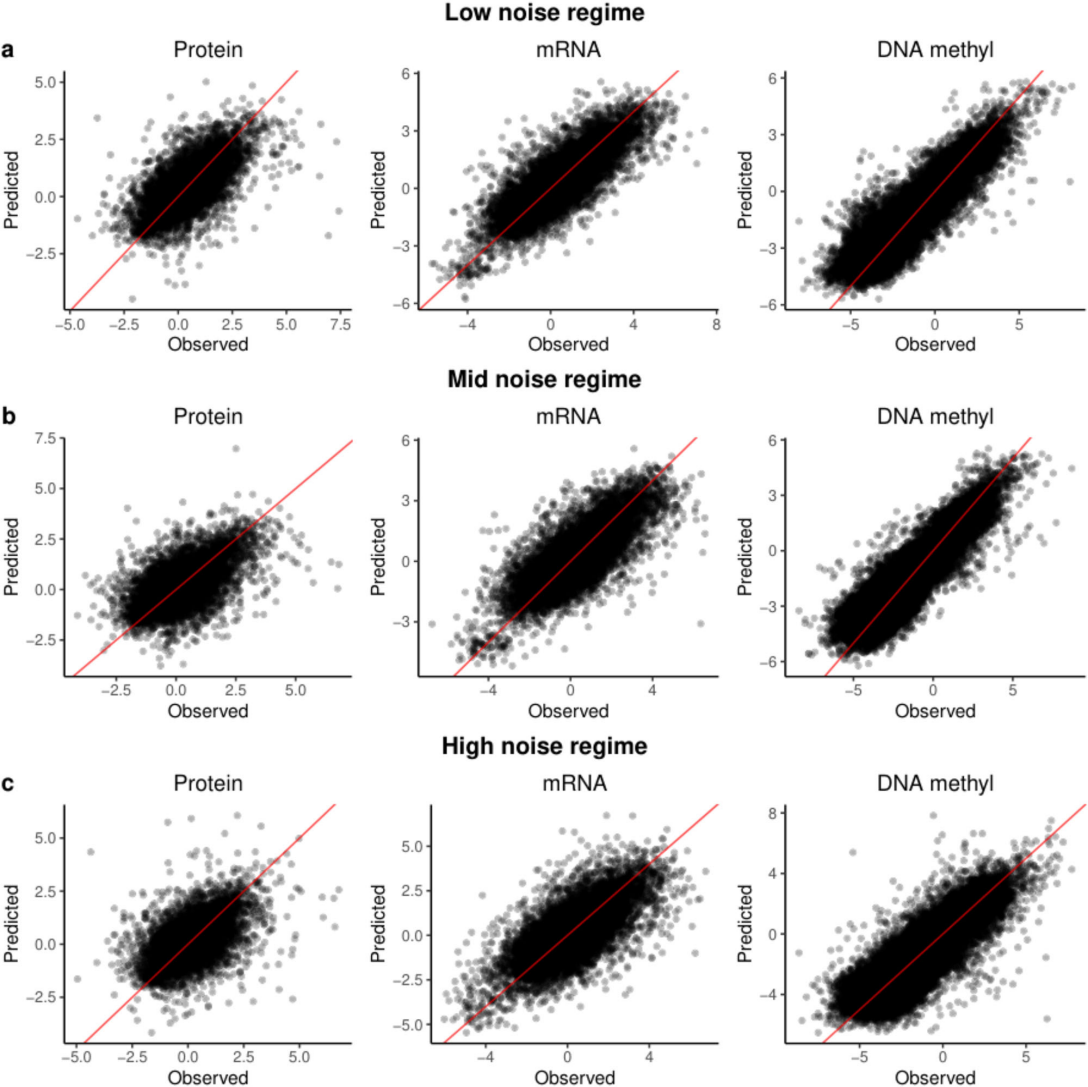
Reconstruction performance. Scatter plots of the reconstructed/predicted value given by MIND vs the observed masked during the training phase at overlap ratio *α* = 0.7. Each point corresponds to one feature in a single sample. **a** Low noise regime. **b** Mid noise regime. **c** High noise regime. Source data for this figure is available in the Source Data file.

For prediction, we used the generated embeddings to reconstruct the mask portion of the synthetic data. For each modality, we compute the Pearson correlations between the reconstructed and observed data (Fig. 3). Here, we can only compare these results with those of the VAE-based JASMINE and MOVE, since network-based IntegrAO and MSNE do not offer this feature. Scatter plots of predicted versus observations given by MIND at *α* = 0.7 are reported in Fig. 4. These showed that MIND predictions of the masked data were more accurate than those provided by JASMINE and MOVE. See Supplementary Note 2 and Supplementary Tables 3–8 for additional ablation studies.

### Pan-cancer prediction tasks

We next applied our proposed method to mRNA-seq, miRNA-seq, DNA methylation, copy number variation (CNV) and functional proteomics reverse phase protein array (RPPA) data from The Cancer Genome Atlas (TCGA)^19^. We investigated 17 data sets from TCGA, which show different degrees of missingness. The sizes of the multi-omics datasets and details on data pre-processing can be found in Methods. In this example, we fix the dimension of latent embedding of our proposed method, IntegrAO, and MSNE to 64. Since the length of JASMINE’s output embeddings has to be a multiple of *M* + 1 where *M* is the total number of modalities, we set its dimension to 66 for a fair comparison. Similar to the synthetic example, we compared MIND with IntegrAO, JASMINE and MSNE on three tasks: survival prediction, cancer type classification, and multi-omics data reconstruction.

**Fig. 5:**
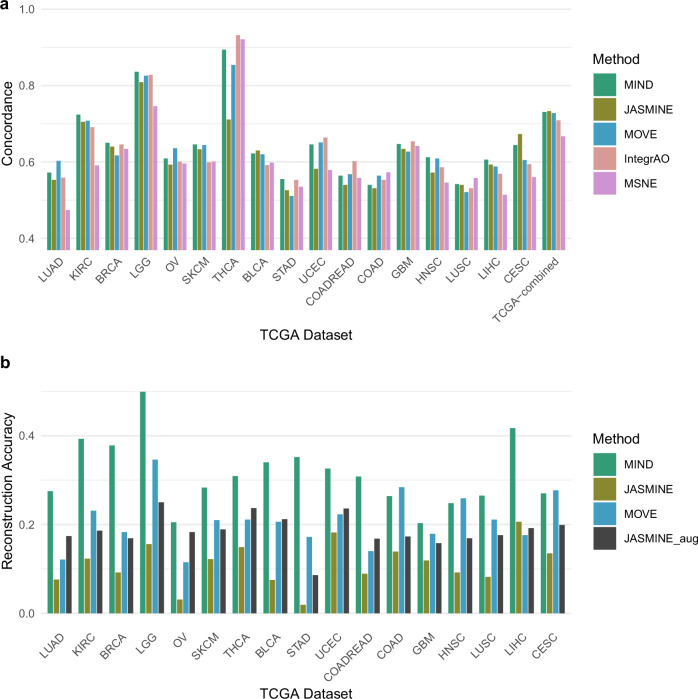
Results of the TCGA dataset. **a** C-index estimated using 5-fold CV for each of the individual TCGA dataset and the combined. For this task, penalised Cox’s proportional hazard’s models are trained to predict survival status of patients using patient information (age, gender) and embeddings given by different methods. **b** Pearson correlations between reconstructed and observed values averaged over all five modalities for each of the individual TCGA dataset. The combined reconstruction accuracy is reported in Fig 6b. JASMINE_aug_ represents the augmented JASMINE model with 64 dimensional modality-specific embeddings. Source data for this figure is available in the Source Data file.

We first investigated the individual datasets. For the survival prediction task, we fit a Coxnet survival model^25^ using both the embeddings generated by each model and patients’ age and gender as feature vectors to predict the survival status of the patients for each dataset. We use the C-index^26^ as a performance metric. For each method and dataset, the C-index calculated using 5-fold CV is reported in Fig. 5a. MIND ranked first or second best for survival prediction for 6/17 and 7/17 cancer types respectively compared to IntegrAO (4/17 and 3/17), JASMINE (3/17 and 1/17), MOVE (2/17, 6/17) and MSNE (2/17 and 1/17). On the combined datasets, the C-index score for MIND was 0.731 compared to 0.733 for JASMINE and 0.728 for MOVE suggesting that the latter benefit from larger sample numbers for improved survival prediction.

We then compared the reconstruction performance of MIND with JASMINE and MOVE (recall that IntegrAO and MSNE do not have this feature). Here, for each modality of each dataset, we first randomly mask 10% of its data subject to the constraint that, for every dataset, each patient must be present in at least one modality of the resulting masked multiomics dataset. We then train the models using the masked datasets, reconstruct the masked data using the learned embeddings, and compare the reconstructed values with the observed values. Since the output embeddings of JASMINE are obtained by concatenating *M* modality-specific embeddings and a global embedding, an embedding vector of length 66 means that each of its modality-specific embedding only has length 11, which could be too stringent to accurately reconstruct data from each modality. We therefore additionally fit an augmented JASMINE such that each individual embedding has length 64 (i.e., the final embedding has length 64(*M* + 1) = 384).

For each modality of each dataset, we computed the Pearson correlation between the reconstructed and observed data. We report the averaged Pearson correlation of all five modalities in Fig. 5b. This shows that correlations between MIND reconstructions of the masked data were more accurate than those provided by both variants of JASMINE and MOVE.

In addition to individual datasets, we also combined the 17 TCGA datasets, and apply MIND to the resulting merged dataset consisting of 8567 patients and 16 cancer types (COAD is a subset of the COADREAD dataset). See Methods for details of the merged dataset.

**Fig. 6:**
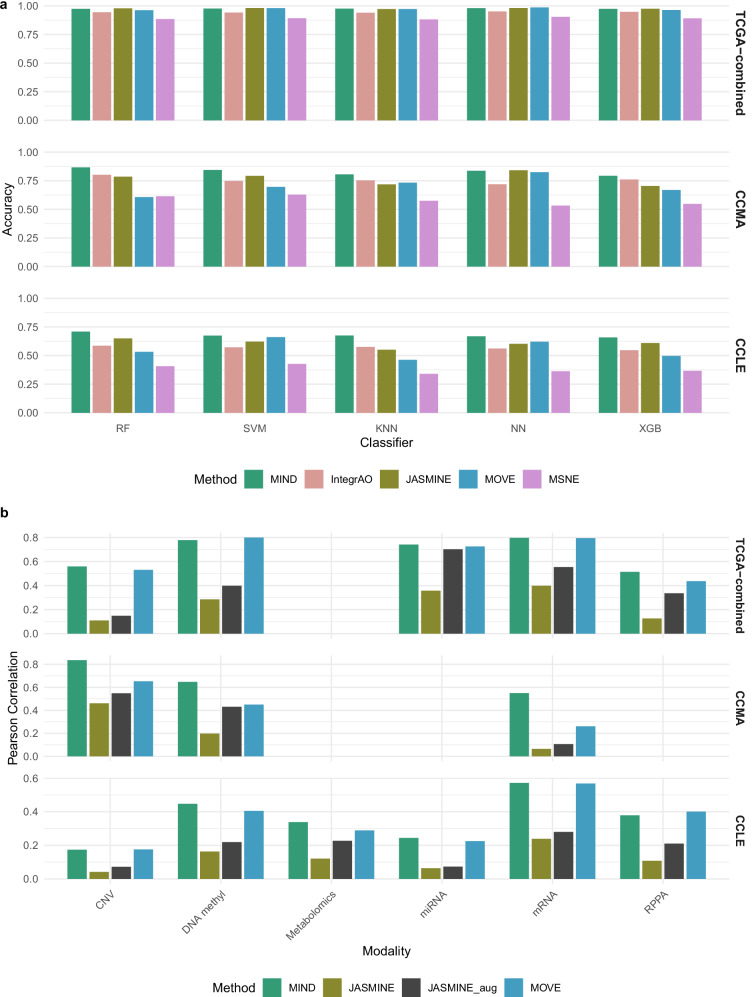
Classification and reconstruction accuracies. **a** Cancer type classification. Classification accuracy estimated using 5-fold CV across TCGA, CCMA, and CCLE datasets and different classifiers (Random Forest, Support Vector Machine, K-Nearest Neighbours, Neural Network and XGBoost). **b** Reconstruction Accuracy. Pearson correlations between reconstructed and observed values of the individual modalities. JASMINE_*aug*_ represents the augmented JASMINE model with 64 dimensional modality-specific embeddings. TCGA-combined shows better accuracies than those of individual datasets in Fig. 5b due to the fact that the variances of data between individual cancer type datasets are much larger than those within individual datasets. Source data for this figure is available in the Source Data file.

We trained classifiers (Random Forest (RF), Support Vector Machine (SVM), K-Nearest-Neighbours (KNN), Neural Network (NN) and XGBoost (XGB)) to predict cancer types using embeddings generated by different methods. Classification accuracies estimated by 5-fold CV are reported in Fig. 6a showing that MIND and JASMINE derived embeddings gave strongest classification results. For all classifiers, the classification accuracy is estimated using the default xgboost^24^ (for XGBoost) or scikit-learn^27^ (for all other classifiers) implementations.

For each of the five modalities, we followed the same masking approach as before, and compared MIND’s ability to reconstruct the missing data to those of JASMINE and MOVE. The Pearson correlations between the reconstructed and observed values in the merged dataset are reported in Fig. 6b show MIND had the strongest overall reconstruction performance.

### MIND can identify clinically relevant subtypes

We next examined the ability of MIND to identify clinically meaningful subtypes through its embeddings using the TCGA BRCA dataset as a representative case study for which extensive work has already been previously conducted to characterise breast cancer molecular subtypes.

**Fig. 7:**
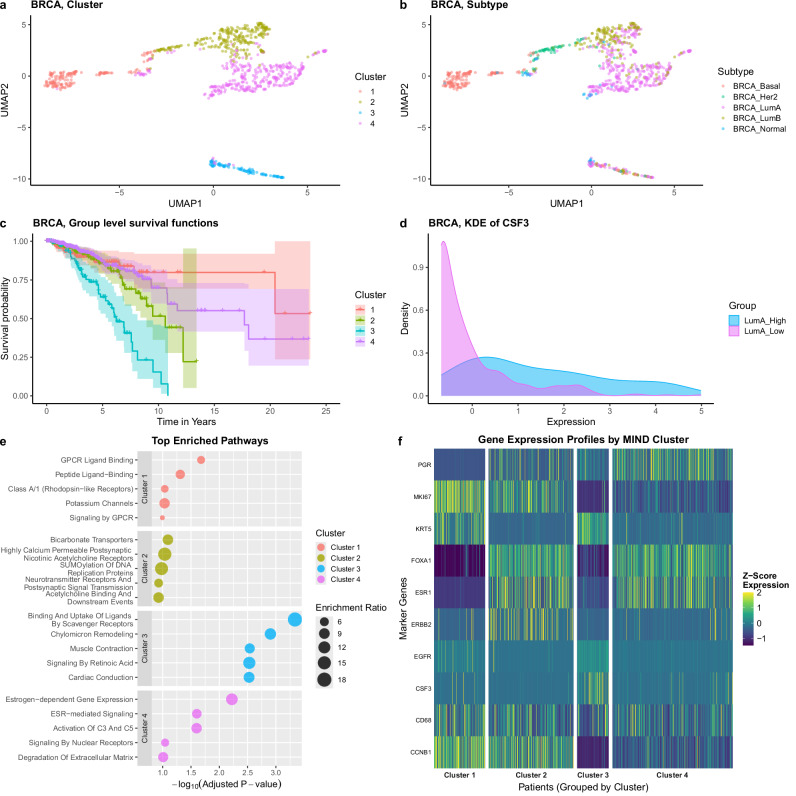
Clustering of BRCA. **a** UMAP of embeddings learnt by MIND, coloured by clustering configuration identified by K-means with *K* = 4. **b** UMAP of embeddings learnt by MIND, coloured by cancer subtypes. **c** Estimated group level survival curve for each cluster in (**a**). **d** KDE of expression level of CSF3 for BRCA-LumA patients belonging to Cluster 3 and 4 in (**a**) respectively. **e** Top five enriched pathways with $-{\log }_{10}({p}_{adj})$ and enrichment ratios for each of the clusters identified by MIND. *p*_*adj*_s are computed using one sided Fisher’s exact test with Benjamini-Hochberg correction. **f** Heat map of standardised mRNA expressions of a subset of marker genes of patients in BRCA dataset. Source data for this figure is available in the Source Data file.

MIND achieved the strongest separation in survival risk for a four-cluster configuration (Fig. 7a, Supplementary Fig. 1) which highlighted four distinct low to high-risk survival profiles (Fig. 7c).

We compared these MIND clusters to the PAM50 molecular subtypes assignments for these tumours (Fig. 7b). We observed that some PAM50 BRCA-LumA and BRCA-Normal tumours were grouped into a single MIND cluster (No. 4). Prior work has shown that the BRCA-Normal subtype likely reflects BRCA-LumA tumours with low tumour purity^12,28^, with subsequent analyses reporting that 67.5% of BRCA-Normal tumours are reclassified as BRCA-LumA following appropriate adjustment^29^. MIND cluster No.2 saw merging of BRCA-LumB and BRCA-Her2, which have been shown to converge on a shared proliferation-associated gene expression programme^30^, and approximately 30% of clinically BRCA-Her2 tumours exhibit a BRCA-LumB intrinsic profile^31^. MIND cluster No.1 corresponds to a basal subtype. We report the number of patients within each cluster identified by MIND and each PAM50 molecular subtype in Table 1.

**Table 1:** Number of patients in each PAM50 molecular subtype and clusters identified by MIND

Cluster/Subtype	Basal	Her2+	LumA	LumB	Normal
Cluster 1	155	12	0	1	3
Cluster 2	0	47	93	142	3
Cluster 3	14	9	56	20	7
Cluster 4	1	7	340	32	23

Source data are provided in the Source Data file.

MIND cluster No.3 identified a distinct group of patients, predominantly of subtype BRCA-LumA, which exhibited markedly lower survival probabilities, suggesting a potential high-risk subgroup within BRCA-LumA (Fig. 7c).^32^ identified a high-risk, Luminal-A–dominant breast cancer subtype (C3) with distinct inflammatory characteristics that exhibits elevated expression of granulocyte colony-stimulating factor (G-CSF) accompanied by neutrophil aggregation. To assess whether Cluster No.3 identified by MIND corresponds to this subtype, we examined CSF3 mRNA expression as a proxy for the high G-CSF phenotype. Specifically, we compared CSF3 expression levels between BRCA-LumA patients in Cluster 3 (LumA-High) and those in Cluster 4 (LumA-Low). As shown in Fig. 7d, LumA-High patients exhibited substantially higher CSF3 expression, consistent with the defining characteristic of the C3 subtype. Fig. 7f further showed that patients in Cluster No.3 show higher CSF3 expression than the other clusters.

To characterise the biological relevance of MIND-derived patient clusters, we further performed a gene set enrichment analysis: We first identified differentially expressed genes (DEGs) for each cluster by performing a one-vs-rest comparison using a two-sided *t*-test on the standardised mRNA expression values of the top 2000 most variable genes. Genes were considered potential markers if they exhibited a positive *t*-statistic. We then ranked these genes by their test statistic and performed an over-representation analysis using the Reactome 2022 pathway database^33^ via the Enrichr server^34^ through gseapy library. Pathways with an adjusted *p*-value less than 0.05 were considered statistically significant. Top 5 enriched pathways for each cluster are reported in Supplementary Table 2 and Fig. 7e.

We validated the biological relevance of MIND-derived clusters by examining their enriched molecular pathways. We found that Cluster No. 4 showed significant enrichment for Oestrogen-dependent Gene Expression (*p*_adj_ = 0.006) and ESR-mediated signalling (*p*_adj_ = 0.025). These findings are consistent with the clinical profile of Luminal A breast cancer^35,36^, confirming that MIND correctly identifies the hormone-driven Luminal A phenotype which is associated with better survival outcomes. In contrast, Cluster No. 3, which we hypothesised corresponded to the high risk, inflammatory C3 subtype, showed significant enrichment for Scavenger Receptor Ligand Binding and Uptake (*p*_adj_ < 0.001). Scavenger receptors are predominantly expressed in macrophages and other innate immune cells^37^, suggesting that Cluster No. 3 is also characterised by distinct immune infiltration and inflammatory signalling rather than pure hormonal drivers. This further supports the correspondence between Cluster No. 3 and inflammatory C3 subtype. Additionally, Cluster No. 1, which corresponded to the Basal subtype, showed enrichment for GPCR Ligand Binding (*p*_adj_ = 0.021), a pathway frequently implicated in the aggressive metastatic potential of basal and triple-negative breast cancers^38^, contrasting with the hormone-driven pathways of Luminal tumours. This further confirms the biological relevance of MIND embeddings. Additional investigation on biological relevance can be found in Supplementary Note 1 and Supplementary Figs. 1–2.

Together, these analyses indicate that MIND-derived embeddings can identify plausible molecular subtypes with clinically distinct characteristics.

### Childhood cancer model Atlas

We also applied our proposed method to mRNA-seq, DNA methylation, and copy number variation (CNV) data from the Childhood Cancer Model Atlas (CCMA) dataset^20^. The dataset consists of multiomics data of 182 paediatric cancer cell lines representing 15 different types of childhood tumour. Details regarding data preprocessing can be found in Methods.

We compare the proposed method with IntegrAO, JASMINE, MOVE and MSNE on cancer type classification and multiomics data reconstruction. Similarly to the previous examples, we fix the embedding size to 64. The classification accuracies estimated by 5-fold CV are reported in Fig. 6a with MIND showing greater classification accuracy than the other methods across all classifiers. For each of the three modalities, we also reconstructed masked data and computed the Pearson correlations between the reconstructed and observed values. Figure 6b shows that for all three modalities (mRNA, methylation and copy number), MIND demonstrated the best reconstruction performance.

### Cancer cell line encyclopaedia

We applied MIND to the Cancer Cell Line Encyclopaedia (CCLE)^21^ dataset. The dataset includes mRNA-seq, DNA methylation, copy number variation (CNV), microRNA, proteomics reverse phase protein array (RPPA), and metabolomics data for 1461 cell lines. We benchmarked MIND against other methods on cancer type classification and data reconstruction as before. Results are reported in Fig. 6 showing the improved MIND classification and reconstruction performance compared to other methods.

#### Integrating multimodal data using MIND improves cancer classification accuracy

We finally used the CCLE dataset as an example to illustrate how integrating multimodal data improves MIND’s performance on cancer type classification. We compared the performance of cancer type classification accuracies of MIND embeddings estimated based on the multimodal dataset and each of the individual ones (RNA, DNA methylation, CNV, miRNA, RPPA, and Metabolomics). For each modality, we train two MIND model independently using the single modality dataset and the corresponding multimodal data of the patients present in that modality. We found the accuracy of the multimodal MIND is consistently higher than those trained on individual datasets, confirming that integrating multimodal data improves the performance of the learnt embeddings on the downstreaming classification task (Table 2). Note that for embeddings trained on individual datasets, the classification accuracy are estimated based on all the patients present in that modality. In contrast, for embeddings trained on multimodal dataset, the classification accuracy is estimated from all available patients in the cohort. As a result, incorporating multimodal data also allows us to analyse a larger set of patients using a single representation learning model.

**Table 2:** Cancer type classification accuracy using multiomics vs single modality data for each of the available modalities in CCLE dataset

Modality/Classifier		RF	SVM	KNN	NN	XGB
RNA	Multi	**0.701**	**0.673**	**0.686**	**0.670**	**0.652**
	Single	0.678	0.645	0.674	0.654	0.622
DNA methyl	Multi	**0.706**	**0.683**	**0.674**	**0.674**	**0.658**
	Single	0.594	0.582	0.559	0.579	0.567
CNV	Multi	**0.699**	**0.674**	**0.686**	**0.676**	**0.649**
	Single	0.210	0.187	0.154	0.205	0.200
miRNA	Multi	**0.700**	**0.673**	**0.681**	**0.664**	**0.652**
	Single	0.338	0.306	0.292	0.304	0.320
RPPA	Multi	**0.703**	**0.694**	**0.675**	**0.665**	**0.649**
	Single	0.482	0.477	0.442	0.433	0.446
Metabolomics	Multi	**0.710**	**0.686**	**0.676**	**0.664**	**0.650**
	Single	0.180	0.158	0.136	0.147	0.177

For each modality-classifier pair, the best is in **boldface**. Source data are provided in the Source Data file.

## Discussion

Our proposed data integration approach, MIND, adopts a multimodal VAE architecture to accommodate incomplete multimodal data sets. MIND advances the broader field of multimodal machine intelligence in two ways. First, it demonstrates how data-driven priors, inspired by manifold learning, can be systematically incorporated into generative latent variable models to enhance representation quality under incomplete observations. The data-driven prior in MIND injects intrinsic clustering structures present in different modalities into the individual-level embeddings in an explicit and interpretable fashion, encouraging the learnt embeddings to maximally preserve the clustering information from the observations with arbitrary missing patterns. Second, MIND achieves this with a lightweight architecture that scales linearly with the number of modalities, contrasting with the quadratic complexity of competing designs.

Using multi-omics, we demonstrate state-of-the-art performance in experiments that show that MIND consistently outperforms or achieves performance on par with existing VAE- and network-based state-of-the-art methods on both supervised and unsupervised downstream tasks. This suggests that MIND can better extract biologically meaningful information from incomplete multiomics data. Furthermore, we also demonstrate that MIND consistently outperforms competing methods in terms of predicting unseen data from learnt patient embeddings. This further confirms that MIND is capable of accurately identifying and capturing characteristics of different patients groups in diverse biological contexts.

Together, these contributions position MIND not only as a practical tool for omics but also as a general methodological advance in multimodal generative modelling.

While MIND demonstrates robust performance in integrating incomplete multi-omics data, several limitations remain. First, although MIND effectively addresses the challenge of objects with missing data modalities, it currently relies on preprocessing steps, such as k-NN imputation, to handle missing values within a data modality. This two-step approach creates a disconnect between the imputation of feature-level missingness and the learning of patient-level embeddings. See also Supplementary Note 4 and Supplementary Tables 9–10 for a discussion on the impact of feature-level imputation. A valuable direction for future work would be to extend the probabilistic framework of MIND to natively handle both types of missingness simultaneously via e.g., multiple imputation^39^. By modelling missing features within the VAE architecture itself, we could eliminate the reliance on external imputation methods and potentially improve the preservation of biological signals. Second, the current formulation of MIND treats the neighbourhood information derived from different modalities equally through the exponentially tilted prior. However, multi-omics datasets often exhibit varying levels of signal-to-noise ratios and discriminative power across modalities. In scenarios where specific modalities are dominated by technical noise or batch effects, equal weighting may be suboptimal. See also Supplementary Note 3 for a discussion on the impact of batch effects. Future iterations of MIND could incorporate adaptive weighting mechanisms and batch alignment modules to automatically regulate information flow between modalities. This would further enhance the model’s robustness against heterogeneous noise sources inherent in complex biological assays.

Finally, MIND is currently designed as a “static" integration method, optimised for learning informative embeddings from a specific patient cohort rather than predicting missing modalities for completely new, out-of-sample patients. Direct application of the trained model to new samples carries the risk of performance degradation due to distribution shifts between the training cohort and the new data. To address this, a promising future direction is the development of a dedicated prediction module capable of explicitly modelling and correcting for distribution shifts. Such an extension would enable statistically principled predictions for new patients, significantly broadening the clinical applicability of the framework for tasks such as precision medicine and diagnostic classification.

In this paper, the evaluation of MIND relies on cancer cohorts (TCGA, CCLE and CCMA). This choice is primarily driven by the availability of high-quality, publicly available multi-omics data with well-defined clinical outcomes. Conceptually, there is no inherent reason why MIND’s architecture, designed to handle heterogeneous noise and complex missing patterns, would be invalid for other therapeutic or clinical areas, and exploring its utility in diverse health conditions remains a priority for our future research.

## Methods

### Data preprocessing

#### Synthetic data

Synthetic data are generated using the InterSim CRAN package^22^. We follow^14^, setting the number of patient *N* = 500 and number of groups *C* = 15. The low noise data are generated using parameters effect = 1.5 and p.DMP = 0.1. The mid noise data are generated using parameters effect = 1 and p.DMP = 0.1. The high noise data are generated using parameters delta.methyl = 1, delta.expr = 1, delta.protein = 0.67 and p.DMP = 0.1.

#### TCGA

For the TCGA example, we leveraged TCGA multi-omics data sets across 17 types of cancer: Head and Neck squamous cell carcinoma (HNSC), Lung squamous cell carcinoma (LUSC), Liver hepatocellular carcinoma (LIHC), Cervical and endocervical cancers (CESC), Lung adenocarcinoma (LUAD), Kidney renal clear cell carcinoma (KIRC), Breast invasive carcinoma (BRCA), Brain Lower Grade Glioma (LGG), Ovarian serous cystadenocarcinoma (OV), Skin Cutaneous Melanoma (SKCM), Thyroid carcinoma (THCA), Bladder urothelial carcinoma (BLCA), Glioblastoma multiforme (GBM), Stomach adenocarcinoma (STAD), Uterine Corpus Endometrial Carcinoma (UCEC), Colorectal adenocarcinoma (COADREAD), and Colon adenocarcinoma (COAD). We obtain mRNA expression, DNA methylation, MicroRNA, protein expression data and relevant patient information from the Broad Institute’s Firehose source data (https://gdac.broadinstitute.org). Copy number variation are obtained from cBioportal (https://www.cbioportal.org). For each dataset, we adopt the data preprocessing steps used in ref. ^14^: we remove columns with more than 20% missing values for each of the modalities. We then impute missing values in the resulting datasets using *k*-nearest neighbour. We stress that the removal and imputation steps are applied only to the missing entries in the data vectors of patients already available in a modality and do not apply to the patients that are not present in a modality (i.e., if a patient is present in a modality, and the corresponding data vector contains missing values, the preprocessing steps will impute those missing values; however, if a patient is not present in a modality, its corresponding data remain completely unknown). Let *N* be the total number of unique patients in the multiomics dataset, and *l*_*m*_ be the feature dimension of modality *m*. One could interpret the imputed data of modality *m* as a *N* × *l*_*m*_ matrix where the *i*th row is completely missing if the *i*th patient is not present in modality *m*. For copy number variation, mRNA expression, and DNA methylation data, we select the top 2000 most variable features. Data from all modalities are then standardised so that each feature dimension has mean 0 and standard deviation 1. The combined TCGA dataset is processed in the same way.

#### CCMA

The CCMA dataset consists of three modalities: RNA-sequencing, DNA methylation, and copy number variation. The CCMA dataset and relevant patient information are obtained from the web portal (https://data.mendeley.com/datasets/rnfs539pfw/3). Missing values are imputed using *k*-nearest neighbour. For RNA-sequence and DNA methylation data, we select the top 2000 most variable features. All data are then standardised so that each feature dimension has mean 0 and standard deviation 1.

#### CCLE

The CCLE dataset consists of six modalities: RNA-sequencing, DNA methylation, copy number variation, MicroRNA, protein expression data and metabolomics data. The CCLE dataset and relevant patient information are obtained from the web portal (https://depmap.org/portal/). For RNA-sequence and DNA methylation data, we select the top 2000 most variable features. All data are then standardised so that each feature dimension has mean 0 and standard deviation 1.

### Mathematical model

Our proposed method can be interpreted as a multimodal variational autoencoder. Before we give the details of the proposed model, we first fix the notations. Let *M* be the total number of modalities. Let *N* be the total number of individual patients. Let [*N*] = {1, . . . , *N*}, [*M*] = {1, . . . , *M*}. Let ${X}_{n}^{m}$ be the data for the *m*th modality of the *n*th patient. For each patient *n*, denote ${A}_{n}=\{m:m\in [M],{X}_{n}^{m}\,{{\rm{is\; available}}}\}$ the index set of modalities in which the data of the *n*th patient are available. Similarly, define ${B}^{m}=\{n:n\in [N],{X}_{n}^{m}\,{{\rm{is\; available}}}\}$ be the index set of available patients for each modality. Denote ${{{\bf{X}}}}^{m}={\{{X}_{n}^{m}\}}_{n\in {B}^{m}}$ the observed data from the *m*th modality. Denote ${{{\bf{X}}}}_{n}={\{{X}_{n}^{m}\}}_{m\in {A}_{n}}$ the collection of observed data associated with the *n*th patient. Denote ${{{\bf{X}}}}_{obs}={\{{{{\bf{X}}}}^{m}\}}_{m=1}^{M}={\{{{{\bf{X}}}}_{n}\}}_{n=1}^{N}$ the full multi-omics dataset. In this paper, we assume ${X}_{n}^{m}\in {{\mathbb{R}}}^{{d}_{m}}$ for all *n* ∈ [*N*], *m* ∈ [*M*] for sake of clarity. Extension to e.g., count data is straightforward.

We assume that each patient *i* is associated with a latent embedding ${Z}_{i}\in {{\mathbb{R}}}^{{d}_{l}}$ drawn from some unknown distribution. Our goal is to learn the distributions of patient-specific *Z*_*i*_s from the observed **X**_*obs*_. In the following sections, we first discuss the encoder architecture and the choice of prior and posterior distributions on embeddings, then give the training and inference procedure of the proposed model.

### Encoder architecture

For each modality *m* ∈ [*M*], let ${E}_{{\theta }_{m}}:{{\mathbb{R}}}^{{d}_{m}}\to {{\mathbb{R}}}^{2{d}_{l}}$ be a modality-specific encoder governed by the parameter vector *θ*_*m*_ that maps ${X}_{n}^{m}$, *n* ∈ [*N*] to the latent space. Denote ${h}_{n}^{m}={E}_{{\theta }_{m}}({X}_{n}^{m})$ and ${\mu }_{n}^{m},{s}_{n}^{m}$ the first and last *d*^*l*^ entries of ${h}_{n}^{m}$, respectively. Denote ${{\boldsymbol{\theta }}}={\{{\theta }_{m}\}}_{m=1}^{M}$ the collection of encoder parameters. Since our goal is to learn patient level embeddings *Z*_*n*_, we need to further aggregate the modality-specific ${\{{\mu }_{n}^{m},{s}_{n}^{m}\}}_{m\in {A}_{n}}$ encoded by ${E}_{{\theta }_{m}}{{\rm{s}}}$, and pass the information to *Z*_*n*_. In this paper, we choose to bridge patient level *Z*_*n*_ and modality-specific ${\{{\mu }_{n}^{m},{s}_{n}^{m}\}}_{m\in {A}_{n}}$ for each patient *n* by setting the VAE posterior

1 $${q}_{{{\boldsymbol{\theta }}}}({Z}_{n}| {{{\bf{X}}}}_{n})={{\mathcal{N}}}\left({Z}_{n};\frac{1}{| {A}_{n}| }\mathop{\sum }\limits_{m\in {A}_{n}}{\mu }_{n}^{m},\,\,{{\rm{diag}}}\,\left(\exp \left(\frac{1}{| {A}_{n}| }\mathop{\sum }\limits_{m\in {A}_{n}}{s}_{n}^{m}\right)\right)\right),$$

where diag(*z*) represents a diagonal matrix with diagonal elements equal to *z* and off-diagonal elements 0, and exp(z) denotes element-wise exponential. In principle, one could consider more sophisticated aggregation functions such as Set Transformer^40^ instead of simple averaging. However, we found that it gave satisfactory results in all numerical examples. We therefore do not investigate other alternatives in this paper for simplicity.

### Prior distribution on embeddings

So far we have discussed encoder architecture and posteriors of the embeddings. In this section, we discuss the choice of prior on *Z*_*n*_s. In this paper, we use a partially data-driven prior on *Z*_*n*_s to inject the neighbouring structure information into the embeddings. Our choice of prior is inspired by the variational formulation of *t*-SNE^41^ given in ref. ^42^. Let ${{\bf{Z}}}={\{{Z}_{i}\}}_{i=1}^{N}$ and ${{\mathcal{D}}}({{\bf{Z}}})\in {{\mathbb{R}}}^{N\times N}$ be the pairwise similarity matrix of embeddings **Z** where each entry ${{{\mathcal{D}}}}_{i,j}({{\bf{Z}}})=\frac{1}{1+| | {Z}_{i}-{Z}_{j}| {| }_{2}^{2}}$. For each modality *m* ∈ [*M*], let ${{{\mathcal{D}}}}^{m}\in {{\mathbb{R}}}^{| {B}^{m}| \times | {B}^{m}| }$ be the *t*-SNE’s sparse data affinity matrix obtained by applying *t*-SNE to **X**^*m*^. Let ${{{\mathcal{D}}}}^{m}({{\bf{Z}}})\in {{\mathbb{R}}}^{| {B}^{m}| \times | {B}^{m}| }$ be a sub-matrix of ${{\mathcal{D}}}({{\bf{Z}}})$ whose rows and columns are selected to match ${{{\mathcal{D}}}}^{m}$. Let ${\bar{{{\mathcal{D}}}}}^{m}({{\bf{Z}}}),{\bar{{{\mathcal{D}}}}}^{m}$ be the normalised and vectorised versions of ${{{\mathcal{D}}}}^{m}({{\bf{Z}}}),{{{\mathcal{D}}}}^{m}$, respectively (i.e., an affinity matrix ${{{\mathcal{D}}}}^{m}$ is first normalised by dividing each entry in ${{{\mathcal{D}}}}^{m}$ by the sum of all entries of ${{{\mathcal{D}}}}^{m}$, then flattened into a ∣*B*^*m*^∣^2^ × 1 vector by stacking all columns of the normalised matrix. This operation turns an affinity matrix ${{{\mathcal{D}}}}^{m}$ into a probability vector that sums to 1. Denote *I*_*p*_ a *p* × *p* identity matrix. We define the exponential-tilted prior on **Z** as

2 $$p({{\bf{Z}}})\propto \mathop{\prod }\limits_{n=1}^{N}{{\mathcal{N}}}\left({Z}_{i};{{\bf{0}}},{I}_{{d}_{l}}\right)\times \exp \left(-\mathop{\sum }\limits_{m=1}^{M}KL\left({\bar{{{\mathcal{D}}}}}^{m}| | {\bar{{{\mathcal{D}}}}}^{m}({{\bf{Z}}})\right)\right).$$

Note that ${{\rm{KL}}}\left({\bar{{{\mathcal{D}}}}}^{m}| | {\bar{{{\mathcal{D}}}}}^{m}({{\bf{Z}}})\right)$, the KL divergence between two categorical distributions specified by probability vectors ${\bar{{{\mathcal{D}}}}}^{m},{\bar{{{\mathcal{D}}}}}^{m}({{\bf{Z}}})$, respectively, is nonnegative by definition. This ensures that *p*(**Z**) is still a valid probability distribution whose probability density is known up to a multiplicative constant. Compared to i.i.d. isotropic Gaussian priors, our choice of *p*(**Z**) additionally encourages different subsets of **Z** to cluster in a way similar to the neighbouring structures of **X**^*m*^s, which are encoded as *t*-SNE’s data affinity matrices. This additional information helps the patient level embeddings **Z** better capture the cluster structure from **X**_obs_, and promote information sharing in the latent space. In this paper, the affinity matrices ${{{\mathcal{D}}}}^{m}{{\rm{s}}}$ are computed using *t*-SNE with the perplexity parameter set to 30, as this choice gives satisfactory results in all numerical examples.

MIND relies on the assumption that local manifold structure (i.e., nearest neighbourhood relationships) learnt from raw multi-omics data preserves significant biological signals. This neighbourhood structure learnt from raw data is not perfect since it can be contaminated by e.g., inherent noise and technical artifacts. The synergy between the *t*-SNE-driven prior and the Multimodal VAE architecture in MIND is specifically designed to mitigate this issue: The exponentially tilted prior incorporates (imperfect) neighbourhood structure from the raw data, and guides the latent space to respect that local topology of the patients, preventing the VAE from learning purely reconstruction-guided embeddings that ignores biological similarity, while the VAE component prevents the embeddings from simply memorising the noisy *t*-SNE projections if those projections contradict the underlying feature patterns learnt from reconstruction. Therefore, the *t*-SNE-informed prior does not dictate the result alone. Instead, it acts as a topological reference point (i.e., a soft constraint), while the VAE allows the model to deviate from the imperfect topological structure identified by *t*-SNE from the raw data where necessary to ensure reconstruction accuracy.

### Training and inference

We discuss here the training and inference procedures of the proposed model. Let ${G}_{{\phi }_{m}}:{{\mathbb{R}}}^{{d}_{l}}\to {{\mathbb{R}}}^{{d}_{m}}$ be the modality-specific decoder governed by the parameter vector *ϕ*_*m*_ for each *m* ∈ [*M*]. Let ${{\boldsymbol{\phi }}}={\{{\phi }_{m}\}}_{m=1}^{M}$ be the collection of decoder parameters. The training procedure of the proposed model is similar to a VAE^43^: Denote ${q}_{{{\boldsymbol{\theta }}}}({{\bf{Z}}}| {{{\bf{X}}}}_{obs})={\prod }_{n=1}^{N}{q}_{{{\boldsymbol{\theta }}}}({Z}_{n}| {{{\bf{X}}}}_{n})$ the joint posterior distribution of **Z**. Suppose ${p}_{{{\boldsymbol{\phi }}}}({X}_{n}^{m}| {Z}_{n})={{\mathcal{N}}}({X}_{n}^{m};{G}_{{\phi }_{m}}({Z}_{n}),{I}_{{d}_{m}})$, the evidence lower bound of the proposed model takes the form

3 $${{\rm{ELBO}}}({{\boldsymbol{\theta }}},{{\boldsymbol{\phi }}};{{{\bf{X}}}}_{obs})=	 -\frac{1}{2}\mathop{\sum }\limits_{n=1}^{N}\mathop{\sum }\limits_{m\in {A}_{n}}{E}_{{Z}_{n} \sim {q}_{{{\boldsymbol{\theta }}}}(\cdot | {{{\bf{X}}}}_{n})}\left(| | {X}_{n}^{m}-{G}_{{\phi }_{m}}({Z}_{n})| {| }_{2}^{2}\right)\\ 	 -KL\left({q}_{{{\boldsymbol{\theta }}}}({{\bf{Z}}}| {{{\bf{X}}}}_{obs})| | p({{\bf{Z}}})\right),$$

where ${E}_{{Z}_{n} \sim {q}_{{{\boldsymbol{\theta }}}}(\cdot | {{{\bf{X}}}}_{n})}$ means taking expectation w.r.t. *Z*_*n*_ ~ *q*_**θ**_( ⋅ ∣**X**_*n*_). The first and second term of ELBO(***θ***, ***ϕ***; **X**_obs_) are the log likelihood and the KL divergence between the posterior and prior, respectively. Here, we assume ${X}_{n}^{m}$ follows a Gaussian distribution. Extension to other likelihoods is straightforward.

In addition to the standard ELBO of a VAE model, we also incorporate a regularisation term on the encoder outputs ${\{{h}_{n}^{m}\}}_{m\in {A}_{n}}$ for all *n* ∈ [*N*] taking the form

4 $$R({{\boldsymbol{\theta }}};{{{\bf{X}}}}_{{\mathrm{obs}}})=\frac{1}{{d}_{l}}\mathop{\sum }\limits_{n=1}^{N}\mathop{\sum }\limits_{m\in {A}_{n}}\mathop{\sum }\limits_{{m}^{{\prime} } < m}| | {h}_{n}^{m}-{h}_{n}^{{m}^{{\prime} }}| {| }_{2}^{2}.$$

Intuitively speaking, *R*(**θ**; **X**_obs_) penalises pairwise distance between ${\{{h}_{n}^{m}\}}_{m\in {A}_{n}}$ for each patient *n*. Recall that ${\{{h}_{n}^{m}\}}_{m\in {A}_{n}}$ are generated using different modality-specific encoders. We encourage ${\{{h}_{n}^{m}\}}_{m\in {A}_{n}}$ to be close to each other as the data used to generate these quantities are from the same patient, and we want the modality-specific encoders ${E}_{{\theta }_{m}}$ to be aware of this cross-modality connection.

The resulting loss function of the proposed model is

5 $$L({{\boldsymbol{\theta }}},{{\boldsymbol{\phi }}};{{{\bf{X}}}}_{{\mathrm{obs}}})=-{{\mathrm{ELBO}}}({{\boldsymbol{\theta }}},{{\boldsymbol{\phi }}};{{{\bf{X}}}}_{{\mathrm{obs}}})+\alpha R({{\boldsymbol{\theta }}};{{{\bf{X}}}}_{{\mathrm{obs}}}),$$

where *α* > 0 is a hyperparameter controlling the strength of the regulariser *R*(**θ**; **X**_obs_). We set *α* = 0.05 in all numerical examples. The VAE parameters {**θ**, ***ϕ***} are trained using reparameterisation trick^43^ and gradient descent methods. Note that the prior we proposed in Eqn. (2) does not factorise. As a result, we are not able to directly apply standard minibatch stochastic gradient descent. We use a Gibbs sampling style training scheme to address this issue: Denote *BC* the index set of a minibatch of patients. Denote **Z**_*BC*_ the corresponding embeddings and **Z**_−*BC*_ = **Z**⧹**Z**_*BC*_ its complement. For each minibatch stochastic gradient descent step, we replace $KL\left({q}_{{{\boldsymbol{\theta }}}}({{\bf{Z}}}| {{{\bf{X}}}}_{{\mathrm{obs}}})| | p({{\bf{Z}}})\right)$ in Eq (3) by the conditional version $KL\left({q}_{{{\boldsymbol{\theta }}}}({{{\bf{Z}}}}_{BC}| {{{\bf{Z}}}}_{-BC},{{{\bf{X}}}}_{{\mathrm{obs}}})| | p({{{\bf{Z}}}}_{BC}| {{{\bf{Z}}}}_{-BC})\right)$. Since the posterior *q*(**Z**∣**X**_obs_) is factorisable by design, we have

6 $${q}_{{{\boldsymbol{\theta }}}}({{{\bf{Z}}}}_{BC}| {{{\bf{Z}}}}_{-BC},{{{\bf{X}}}}_{{\mathrm{obs}}})={q}_{{{\boldsymbol{\theta }}}}({{{\bf{Z}}}}_{BC}| {{{\bf{X}}}}_{{\mathrm{obs}}})=\mathop{\prod }\limits_{n\in BC}{q}_{{{\boldsymbol{\theta }}}}({Z}_{n}| {{{\bf{X}}}}_{n}).$$

The second term *p*(**Z**_*BC*_∣**Z**_−*BC*_) is equivalent to $p\left(\left\{{{{\bf{Z}}}}_{BC}\right.,{{\rm{sg}}}({{{\bf{Z}}}}_{-BC})\right\}$ in the training process, where sg is the stop-gradient operator (i.e., we treat **Z**_−*BC*_ as fixed by detaching it from the computation graph). Computing the corresponding minibatch log likelihood and regularisation is straightforward. The final minibatch loss is obtained by combing the individual minibatch terms the same way as in Eq (5).

Suppose $\widehat{{{\boldsymbol{\theta }}}},\widehat{{{\boldsymbol{\phi }}}}$ are approximate minimisers of *L*(***θ***, ***ϕ***; **X**_obs_). Then each patient embedding *Z*_*n*_ is represented by the posterior ${q}_{\widehat{{{\boldsymbol{\theta }}}}}({Z}_{n}| {{{\bf{X}}}}_{n})$. We compute the reconstructed or predicted data ${\widehat{X}}_{n}^{m}$ for any *n* ∈ [*N*], *m* ∈ [*M*] by passing either the mean or a sample from ${q}_{\widehat{{{\boldsymbol{\theta }}}}}({Z}_{n}| {{{\bf{X}}}}_{n})$ to the corresponding trained decoder ${G}_{{\widehat{\phi }}_{m}}$. See Fig. 1c for a schematic illustration of the proposed method.

### Running times

Running times of all methods are reported in Table 3. All running times are record on our desktop with an AMD 5700 CPU and a Nvidia RTX2060 GPU. We found that network-based methods (IntegrAO and MSNE) are faster to train. Among VAE based methods, MIND and MOVE have similar training time, while the training time for JASMINE is longer, which is likely due to its additional cross modality modules.

**Table 3:** Running time (in seconds) of different methods on the three datasets

Dataset/Method	MIND	IntegrAO	JASMINE	MOVE	MSNE
TCGA	2.47 × 10^3^	$\underline{9.33\times 1{0}^{1}}$	8.47 × 10^3^	3.35 × 10^3^	**5.45** × **10**^**1**^
CCLE	1.75 × 10^3^	$\underline{5.72\times 1{0}^{1}}$	5.02 × 10^3^	1.69 × 10^3^	**3.18** × **10**^**1**^
CCMA	6.13 × 10^2^	$\underline{2.05\times 1{0}^{1}}$	1.19 × 10^3^	6.46 × 10^2^	**1.69** × **10**^**1**^

Best in **boldface**. Second best is underlined. Source data are provided in the Source Data file.

### Statistics and reproducibility

No statistical method was used to predetermine the sample sizes or allocation. The numerical experiments carried out in this paper can be fully reproduced using the random seed supplied in the code.

### Reporting summary

Further information on research design is available in the Nature Portfolio Reporting Summary linked to this article.

## Supplementary information

Supplementary Information

Description of Additional Supplementary Files

Supplementary Data 1

Reporting Summary

Transparent Peer Review file

## Source data

Source Data

## Data Availability

All datasets and output files can be downloaded from Figshare^44^. Source data are provided with this paper. mRNA expression, DNA methylation, MicroRNA, protein expression data and relevant patient information of the TCGA dataset are obtained from Broad Institute’s Firehose source data (https://gdac.broadinstitute.org). The Copy number variation data are obtained from cBioportal (https://www.cbioportal.org). The CCMA dataset and relevant patient information are obtained from the web portal (https://data.mendeley.com/datasets/rnfs539pfw/3). The CCLE dataset and relevant patient information are obtained from the web portal (https://depmap.org/portal/). A full description of all datasets is provided in Supplementary Data 1. Source data are provided with this paper.

## Code availability

A Python implementation of MIND, as well as codes for reproducing all experiments in the paper, can be found on Github^45^ (https://github.com/hwxing3259/MIND/).
