# Supplementary material for: MIND: multimodal integration with neighbourhood-aware distributions
Source: Nat Commun. 2026 Jun 24;17:7901. doi: 10.1038/s41467-026-74413-1 (PMC13442840; doi:10.1038/s41467-026-74413-1)
Supplement: Supplementary file 1 [file 41467_2026_74413_MOESM1_ESM.pdf]

Supplementary Information
Multimodal Integration with Neighbourhood-aware Distributions

Hanwen Xing [1], Christopher Yau [1, 2, *]

[1] Nuffield Department for Women's and Reproductive Health, University of Oxford, UK.
[2] Health Data Research UK, London, UK.

[*] Corresponding author: christopher.yau@wrh.ox.ac.uk

Supplementary Note 1: MIND gives clinically meaningful embeddings

In addition to survival prediction, we investigated survival differentiation and clinical label enrichment to highlight the clinical relevance and meaningfulness of the embeddings learnt by MIND. For each of the TCGA dataset, we use K-means clustering to cluster the learnt embeddings, and investigate differential survival between the resultant clusters using the log rank test (testing if survival risks are significantly difference across clusters). Similarly to [1], we report the negative log p-value of the log rank test as a metric of differential survival (larger negative log p-value means greater inter-cluster survival risks difference). We then examine the enrichment of six clinical labels, including gender, age at diagnosis, TMN staging (pT, pM, pN), and pathologic stage (total progression), i.e. testing if the distributions of these labels are significantly different across clusters, for datasets whose corresponding labels are available (12 out of 17). The enrichments for discrete and continuous labels were assessed using the χ^2 and Kruskal-Wallis tests, respectively. For each method and cluster configuration, we report the number of enriched clinical labels (i.e. labels whose corresponding adjusted p-value is less than 0.05). Due to the lack of ground truth value of the cluster numbers, we repeat the analysis for a range of cluster numbers from 3 to 10. The results are reported in Figures 1 and 2. MIND showed superior or competitive performance on both survival differentiation and clinical label enrichment for the majority of the TCGA datasets, confirming the clinical relevance of MIND's output embeddings.

Supplementary Note 2: Ablation studies

We investigate the contribution of both the t-SNE prior and the patient level regularisation using the CCLE dataset as an example. Specifically, we fix embedding size to be 64 and perplexity parameter to be 30, and fit four variants of MIND: MIND_{VAE} (without either the t-SNE informed prior or the patient level regularisation), MIND_t (with the t-SNE informed prior but without the patient level regularisation), MIND_r (without the t-SNE informed prior but with the patient level regularisation, $\alpha = 0.05$), and MIND (with both the t-SNE informed prior and the patient level regularisation, $\alpha = 0.05$). (We put isotropic Gaussian prior on the embeddings whenever the t-SNE informed prior is not used.) We found that the inclusion of

both the t-SNE informed prior and the pateint-level regularisation improves the performance of MIND in both classification and reconstruction accuracy, confirming the efficacy of both terms (Table 3, 4).

We have also investigated the impact of the perplexity parameter of t-SNE by fixing the embedding size at 64, and comparing the performance of MIND with perplexity parameter set to $8, 15, 30, 45, 60$. We found MIND is robust under various choices of t-SNE perplexity parameters (Table 5, 6).

Finally, we investigate the impact of embedding sizes. Here we fix the perplexity parameter at 30, and experiment with embedding size $8, 16, 32, 64, 128, 256$. We found that increasing embedding size leads to a diminishing improvement in performance of downstreaming tasks such as classification accuracy, but also a poorer performance on reconstruction accuracy (Table 7, 8). This is not surprising as higher dimensional embeddings have the additional flexibility to capture finer details of the dataset. But this flexibility also makes the model prone to e.g. overtraining that leads to the poor performance on data reconstruction. As a result, we recommend setting a moderate embedding size $d = 64$ to provide sufficient flexibility while preventing over-fitting.

Supplementary Note 3: Impact of batch effects

Bulk omics data such as TCGA, CCLE and CCMA can be susceptible to batch effects, which may influence the neighbourhood structure identified by *t*-SNE, and therefore bias the neighbourhood information and compromise the quality of the final embeddings. For example, site- or lab-specific processing capabilities could introduce a weak correlation with modality availability. However, we emphasize that in the "barcode integration" setting, this correlation is partial and stochastic, rather than systematic: Patients in the same sequencing centre may have different modality availability patterns, while patients in different sequencing centres may share the same availability pattern. Consequently, even if there are correlations between modality availability and sequencing centres, the neighbourhood structures computed within any *single* modality are based on patients from highly heterogeneous processing origins, which naturally dilutes site-specific biases within any single affinity matrix.

Indeed, the highly heterogeneous processing origins can still introduce non-independent noise to the *t*-SNE priors built on the affinity matrices, and potentially bias the learnt embeddings. However, the ablation studies above using the Cancer Cell Line Encyclopedia (CCLE) dataset provided empirical evidence showing that the *t*-SNE prior injects a net-positive biological signal. If the *t*-SNE-derived neighbourhood structures predominantly capture and propagate technical noise rather than biologically meaningful topology, we would expect the baseline model operating *without* the *t*-SNE informed prior (MIND_{VAE}) to match or outperform the full MIND model *with* the *t*-SNE informed prior. Instead, we found that the full MIND model consistently and substantially outperforms MIND_{VAE} in all classification and reconstruction tasks (Supplementary Tables S2 and S3). This demonstrates that the soft-constraint nature of the exponentially tilted prior successfully incorporates meaningful biological structure, and that the VAE reconstruction objective is capable of correcting the embeddings where the raw, noisy topological structure potentially contradicts underlying feature patterns.

Supplementary Note 4: Impact of imputation

We use the CCMA dataset as an example to illustrate the impact of imputation. We benchmark different models on a trimmed version of CCMA such that only features with full data

are included (i.e. all columns containing missing values are removed) in a similar fashion to the imputed case. Results are reported in Table 9, 10. Similar to the imputed case, MIND demonstrated the best performance. We also found that cancer type classification accuracies of all methods were worse than ones based on the imputed data. This is likely due to the fact that informative features were removed in the trimming process. On the other hand, the reconstruction performances were better. But we believe this is because the model is handling a easier reconstruction task (a much smaller dataset), not because it has learnt the underlying biological signal better.

Method	Supervised	Single cell	Mosaic integration	Paired	Integrated embedding
MIND	N	N	N	Y	Y
MOFA	N	N	N	Y	Y
MOGONET	Y	N	N	Y	Y
DLVPM	N	N	N	Y	N
MIDAS	N	Y	Y	Y	Y
GLUE	N	Y	N	N	Y
scMoMaT	N	Y	Y	Y	Y
StabMap	N	Y	Y	Y	Y

Supplementary Table 1: Comparison of multi-omics integration methods.

**Cluster**	**Pathway Term**	**Adj. p-value**
**Cluster 1**	GPCR Ligand Binding	0.021
	Peptide Ligand-Binding Receptors	0.049
	Class A/1 (Rhodopsin-like Receptors)	0.092
	Potassium Channels	0.093
	Signaling By GPCR	0.102
**Cluster 2**	Bicarbonate Transporters	0.081
	Highly Calcium Permeable Postsynaptic Nicotinic Acetylcholine Receptors	0.092
	SUMOylation Of DNA Replication Proteins	0.105
	Acetylcholine Binding And Downstream Events	0.118
	Neurotransmitter Receptors And Postsynaptic Signal Transmission	0.118
**Cluster 3**	Binding And Uptake Of Ligands By Scavenger Receptors	4.6e-04
	Chylomicron Remodeling	0.001
	Cardiac Conduction	0.003
	Signaling By Retinoic Acid	0.003
	Muscle Contraction	0.003
**Cluster 4**	Estrogen-dependent Gene Expression	0.006
	ESR-mediated Signaling	0.025
	Activation Of C3 And C5	0.025
	Signaling By Nuclear Receptors	0.090
	Degradation Of Extracellular Matrix	0.090

Supplementary Table 2: Top Enriched Reactome Pathways for MIND-Derived Clusters. Adj. p-values are computed using one sided Fisher's exact test with Benjamini-Hochberg correction. Source data are provided as a Source Data file.

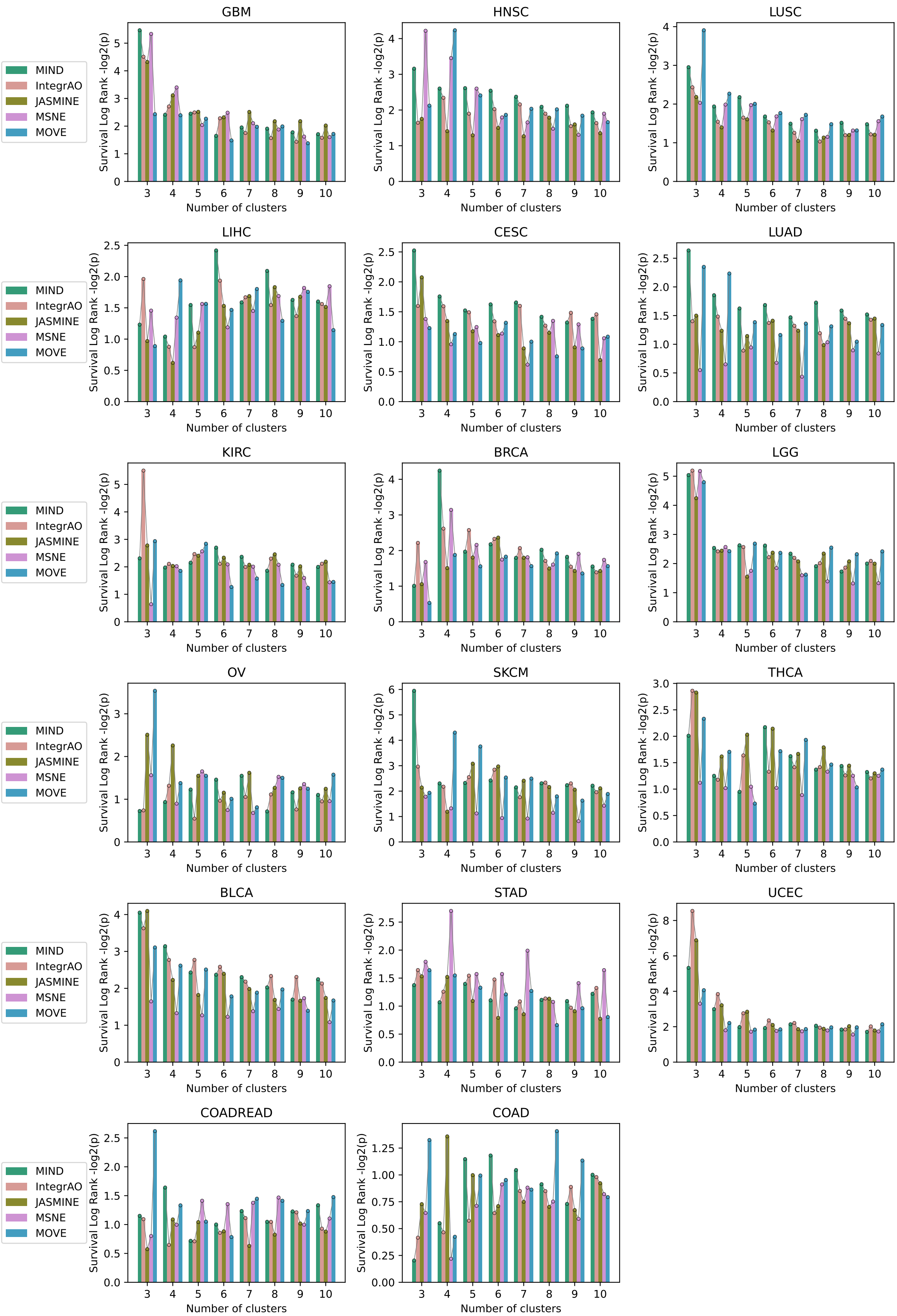

Supplementary Figure 1: Differential survival: Histogram of $-\log(p)$ of the log rank test for each of the TCGA dataset. p-values are computed using log rank test. Source data are provided as a Source Data file.

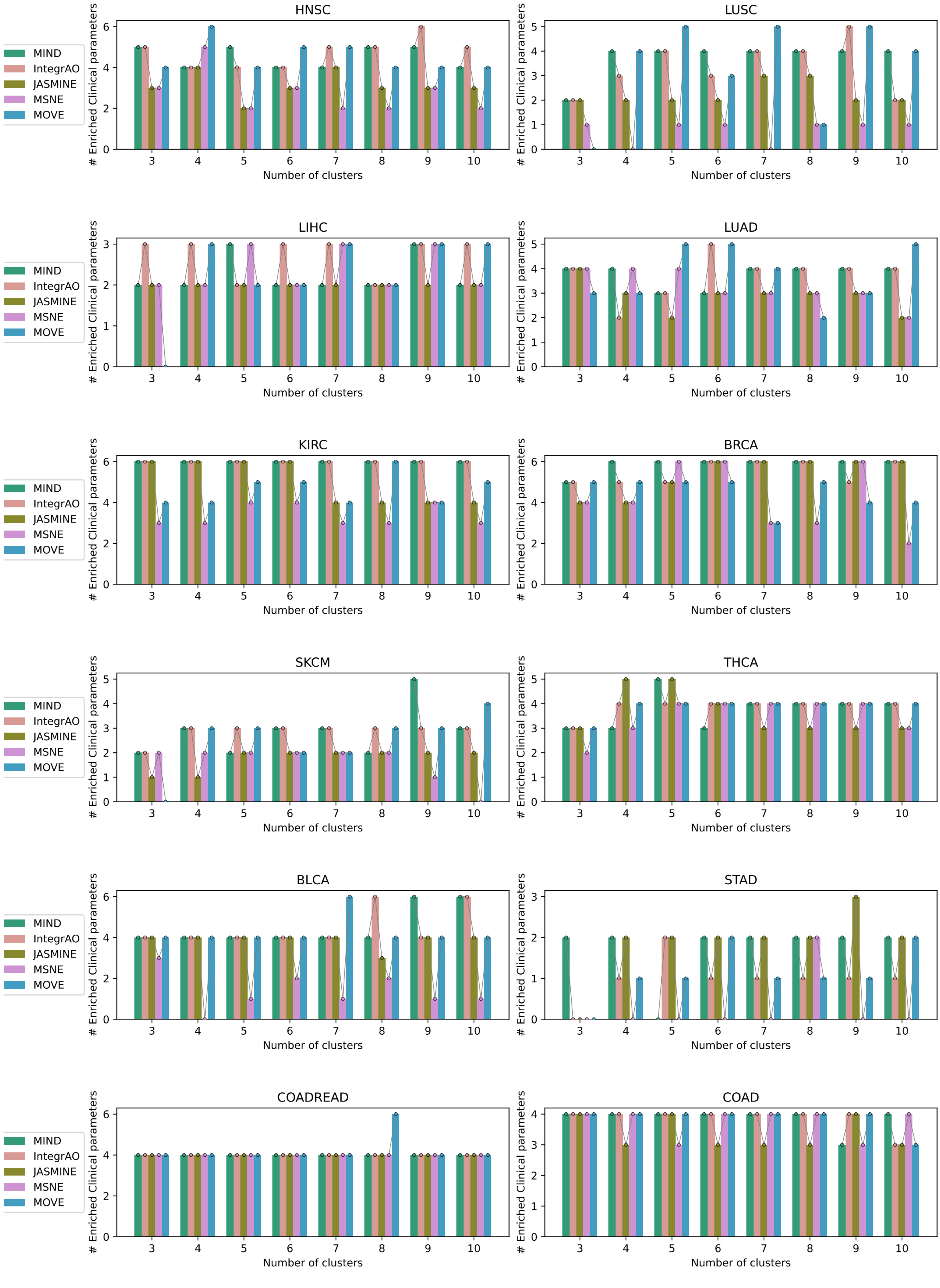

Supplementary Figure 2: Clinical label enrichment: Histogram of number of enriched clinical labels for each of the TCGA dataset. Source data are provided as a Source Data file.

Embedding size / Method	RF	SVM	KNN	NN	XGB
MIND_{VAE}	0.464	0.367	0.252	0.403	0.471
MIND_t	<u>0.678</u>	<u>0.651</u>	<u>0.602</u>	<u>0.591</u>	<u>0.626</u>
MIND_r	0.461	0.436	0.321	0.420	0.468
MIND	**0.709**	**0.674**	**0.675**	**0.668**	**0.659**

Supplementary Table 3: Classification accuracy of different variants of MIND, CCLE. Best in **boldface**. Second best is <u>underlined</u>. MIND_{VAE}: without either the t-SNE informed prior or the patient level regularisation); MIND_t: with the t-SNE informed prior but without the patient level regularisation); MIND_r: without the t-SNE informed prior but with the patient level regularisation, $\alpha = 0.05$; MIND: with both the t-SNE informed prior and the patient level regularisation, $\alpha = 0.05$. Source data are provided as a Source Data file.

Variant/Modality	RNA	DNA methyl	CNA	miRNA	RPPA	Metabolomics
MIND_{VAE}	0.532	0.403	0.103	0.171	0.353	0.253
MIND_t	<u>0.571</u>	<u>0.410</u>	<u>0.128</u>	<u>0.247</u>	<u>0.374</u>	<u>0.304</u>
MIND_r	0.509	0.372	0.094	0.120	0.354	0.205
MIND	**0.572**	**0.447**	**0.174**	**0.244**	**0.379**	**0.338**

Supplementary Table 4: Reconstruction accuracy of different variants of MIND, CCLE. Best in **boldface**. Second best is <u>underlined</u>. MIND_{VAE}: without either the t-SNE informed prior or the patient level regularisation); MIND_t: with the t-SNE informed prior but without the patient level regularisation); MIND_r: without the t-SNE informed prior but with the patient level regularisation, $\alpha = 0.05$; MIND: with both the t-SNE informed prior and the patient level regularisation, $\alpha = 0.05$. Source data are provided as a Source Data file.

Perplexity / Method	RF	SVM	KNN	NN	XGB
8	0.707	<u>0.676</u>	0.670	0.657	**0.682**
15	**0.714**	**0.678**	0.672	**0.669**	<u>0.672</u>
30	<u>0.709</u>	0.674	<u>0.675</u>	<u>0.668</u>	0.659
45	0.706	0.667	0.674	0.665	0.666
60	0.693	0.674	**0.676**	0.662	0.664

Supplementary Table 5: Classification accuracy under different choices of tSNE perplexity parameter, CCLE. Best in **boldface**. Second best is <u>underlined</u>. Source data are provided as a Source Data file.

Perplexity/Modality	RNA	DNA methyl	CNA	miRNA	RPPA	Metabolomics
8	0.556	0.403	0.158	0.237	0.362	0.324
15	0.561	0.438	0.167	0.225	**0.417**	0.289
30	<u>0.572</u>	**0.447**	<u>0.174</u>	0.244	0.379	**0.338**
45	**0.574**	0.428	**0.175**	**0.275**	<u>0.397</u>	0.319
60	0.567	<u>0.440</u>	0.162	<u>0.265</u>	0.395	<u>0.333</u>

Supplementary Table 6: Reconstruction accuracy under difference choices of tSNE perplexity parameter, CCLE. Best in **boldface**. Second best is <u>underlined</u>. Source data are provided as a Source Data file.

Embedding size / Method	RF	SVM	KNN	NN	XGB
8	0.684	0.658	0.665	0.657	0.655
16	0.698	0.668	0.667	0.662	0.658
32	0.702	<u>0.676</u>	0.670	<u>0.667</u>	<u>0.671</u>
64	**0.709**	0.674	**0.675**	**0.668**	0.659
128	<u>0.706</u>	**0.678**	0.661	0.663	0.669
256	0.694	0.666	<u>0.672</u>	0.662	**0.681**

Supplementary Table 7: Classification accuracy under difference choices of embedding dimensions, CCLE. Best in **boldface**. Second best is <u>underlined</u>. Source data are provided as a Source Data file.

Embedding size / Modality	RNA	DNA methyl	CNA	miRNA	RPPA	Metabolomics
8	**0.598**	**0.478**	0.182	**0.251**	0.396	<u>0.338</u>
16	<u>0.576</u>	0.455	**0.183**	0.240	<u>0.400</u>	0.337
32	0.570	<u>0.462</u>	<u>0.181</u>	0.243	**0.409**	**0.342**
64	0.572	0.447	0.174	<u>0.244</u>	0.379	<u>0.338</u>
128	0.540	0.417	0.169	0.229	0.378	0.285
256	0.538	0.392	0.154	0.204	0.359	0.257

Supplementary Table 8: Reconstruction accuracy under difference choices of embedding dimensions, CCLE. Best in **boldface**. Second best is <u>underlined</u>. Source data are provided as a Source Data file.

Classifier / Method	MIND	IntegraO	JASMINE	MOVE	MSNE
RF	**0.830**	<u>0.784</u>	0.762	0.604	0.602
SVM	**0.801**	0.735	<u>0.787</u>	0.685	0.613
KNN	**0.790**	<u>0.755</u>	0.703	0.728	0.564
NN	**0.846**	0.707	<u>0.839</u>	0.821	0.535
XGB	**0.792**	<u>0.746</u>	0.701	0.646	0.532

Supplementary Table 9: Classification Accuracy of the trimmed CCMA dataset. Best in **boldface**. Second best is <u>underlined</u>. *Columns with missing data are removed.* Source data are provided as a Source Data file.

Dataset	Modality / Method	MIND	JASMINE	JASMINE$_{aug}$	MOVE
CCMA$_{trim}$	mRNA	**0.561**	0.069	0.111	<u>0.279</u>
	DNA methyl	**0.649**	0.202	0.455	<u>0.484</u>
	CNV	**0.858**	0.483	0.579	<u>0.681</u>

Supplementary Table 10: Reconstruction Accuracy of the trimmed CCMA dataset. Pearson correlations between reconstructed and observed values of the individual modalities. JASMINE$_{aug}$ represents the augmented JASMINE model with 64 dimensional modality-specific embeddings. For each modality, the best result is highlighted in **boldface**. Second best is <u>underlined</u>. *Columns with missing data are removed.* Source data are provided as a Source Data file.

Supplementary References

[1] Shihao Ma, Andy GX Zeng, Benjamin Haibe-Kains, Anna Goldenberg, John E Dick, and Bo Wang. Moving towards genome-wide data integration for patient stratification with integrate any omics. *Nature Machine Intelligence*, 7(1):29–42, 2025.
