# Supplementary material for: MIND: multimodal integration with neighbourhood-aware distributions
Source: Nat Commun. 2026 Jun 24;17:7901. doi: 10.1038/s41467-026-74413-1 (PMC13442840; doi:10.1038/s41467-026-74413-1)
Supplement: Supplementary file 2 [file 41467_2026_74413_MOESM2_ESM.pdf]

Description of Additional Supplementary Files

Supplementary Data 1: provides a complete inventory of all third-party datasets that were reused in this study. Each row corresponds to one external dataset and lists the dataset name, a short description, the omics modalities and clinical / sample annotations that were retrieved, the cohort size used, the source repository, a URL to the dataset landing page, and the reference for the dataset. All listed resources are publicly available.
