# Supplementary material for: MIND: multimodal integration with neighbourhood-aware distributions
Source: Nat Commun. 2026 Jun 24;17:7901. doi: 10.1038/s41467-026-74413-1 (PMC13442840; doi:10.1038/s41467-026-74413-1)
Supplement: Supplementary file 4 [file 41467_2026_74413_MOESM5_ESM.pdf]

nature portfolio

Peer Review File

MIND: Multimodal Integration with Neighbourhood-aware Distributions

Corresponding Author: Professor Christopher Yau

This file contains all reviewer reports in order by version, followed by all author rebuttals in order by version.

Version 0:

Reviewer comments:

Reviewer #1

(Remarks to the Author)
This manuscript introduces MIND, a method for multimodal data integration in biomedical applications, particularly focusing on multi-omics data analysis. MIND leverages a multimodal variational autoencoder (VAE) with a data-driven prior that uses neighborhood structures to align the latent and data spaces. The authors demonstrate the method's robustness to high missing rates and unbalanced missingness patterns, as well as its performance in low signal-to-noise ratio scenarios. Through benchmark experiments on synthetic and real data, the authors have showed MIND's superiority in various tasks, including survival prediction, cancer type classification and data reconstruction. However, the significance of the work is currently limited by three principal concerns: a lack of demonstrated novelty over existing methods, insufficient discussion of methodological details, and the absence of biologically meaningful insights within the findings. The authors are encouraged to address the following points:

Major comments:
1. The field of multi-omics data integration is well-established, with numerous robust methods already in existence. While the technical performance of MIND is demonstrated, the manuscript currently has not sufficiently demonstrated its conceptual or practical novelty. To clearly delineate MIND's contribution, the authors should provide a more rigorous comparison with a wider set of established methods, such as MOFA (2018) [1], MOGONET (2021) [2], MIDAS (2024) [3] and DLVPM (2025) [4]. Many of these methods, especially those designed for single-cell multi-omics (e.g., GLUE (2022) [5], scMoMaT (2023) [6], StabMap (2024) [7]), are inherently capable of handling bulk omics data integration and already address challenges like data heterogeneity and missingness. It is necessary to describe the MIND's unique contribution more clearly in order to justify its place within the field of methodology.

References:
[1] Argelaguet R, Velten B, Arnol D, et al. Multi-Omics Factor Analysis—a framework for unsupervised integration of multi-omics data sets[J]. Molecular systems biology, 2018, 14(6): e8124. https://doi.org/10.15252/msb.20178124
[2] Wang, T., Shao, W., Huang, Z. et al. MOGONET integrates multi-omics data using graph convolutional networks allowing patient classification and biomarker identification. Nat Commun 12, 3445 (2021). https://doi.org/10.1038/s41467-021-23774-w
[3] He, Z., Hu, S., Chen, Y. et al. Mosaic integration and knowledge transfer of single-cell multimodal data with MIDAS. Nat Biotechnol 42, 1594–1605 (2024). https://doi.org/10.1038/s41587-023-02040-y
[4] Ing, A., Andrades, A., Cosenza, M.R. et al. Integrating multimodal cancer data using deep latent variable path modelling. Nat Mach Intell 7, 1053–1075 (2025). https://doi.org/10.1038/s42256-025-01052-4
[5] Cao, ZJ., Gao, G. Multi-omics single-cell data integration and regulatory inference with graph-linked embedding. Nat Biotechnol 40, 1458–1466 (2022). https://doi.org/10.1038/s41587-022-01284-4
[6] Zhang, Z., Sun, H., Mariappan, R. et al. scMoMaT jointly performs single cell mosaic integration and multi-modal bio-marker detection. Nat Commun 14, 384 (2023). https://doi.org/10.1038/s41467-023-36066-2
[7] Ghazanfar, S., Guibentif, C. & Marioni, J.C. Stabilized mosaic single-cell data integration using unshared features. Nat Biotechnol 42, 284–292 (2024). https://doi.org/10.1038/s41587-023-01766-z

2. In the both Abstract and Introduction sections, the manuscript claims that existing methods often rely on imputation or sample removal, which can lead to information loss, while MIND learns directly from incomplete data via a multimodal VAE. However, when pre-processing datasets such as TCGA and CCMA, the authors first remove features with missing rates

exceeding 20%, and then apply KNN imputation to the remaining missing values. This results in the model being trained on an approximately complete matrix after imputation, rather than on the original complex missing data structure. This approach appears inconsistent with the motivation of this work. It is recommended that the authors retain the original missing data structure in real-data experiments or, at a minimum, include comparative experiments without KNN imputation.

3. The manuscript evaluates MIND's performance on supervised and unsupervised tasks such as survival prediction, cancer type classification, and data reconstruction. However, a significant weakness is the exclusive reliance on quantitative metrics, failing to leverage these results to deliver new biological insights. The authors claim that "MIND can better extract biologically meaningful information from incomplete multi-omics data." However, this claim remains unsubstantiated by any concrete biological interpretation from the results. It is highly recommended that the authors provide analysis addressing the following questions:
a) How do the learned embeddings or the integrated view from MIND help us understand the biological processes in the studied datasets (TCGA, CCMA, and CCLE)? An analysis linking the model's feature representations to established biology is crucial for interpretability.
b) Can MIND generate novel biological hypotheses? The authors should present some specific case studies derived from their analysis. For example, did MIND identify a patient subgroup with distinct molecular features, a corresponding survival difference that might be clinically relevant, or a biologically meaningful regulatory network that was missed by other methods? Can this model accurately identify the biomarkers that contribute most significantly to cancer types?

4. In the second paragraph of the Introduction section, the authors states: "This design principle enables the model to maintain interpretable and biologically meaningful representations in biomedical domains, while also generalising to any multimodal learning setting where incomplete data and heterogeneous noise are pervasive." This is a valuable claim. However, the Results section currently lacks a dedicated analysis to substantiate it. To fully support this statement and enhance the impact of the work, the authors are encouraged to include a specific case study that explicitly demonstrates how the learned representations are interpretable and lead to biologically meaningful insights. For instance, analyzing what the model has learned about specific genes or pathways would be highly compelling.

5. The results in Figure 2 raises an important question regarding the integration behavior of MIND. It is evident that the structures in the individual modality UMAPs (a-c) vary in clarity, with Protein modality (Fig.2a) exhibiting the most distinct separation, while the DNA methylation modality (Fig.2c) appears to contribute less to the final result. The authors should discuss the potential reasons for this observation. For instance, does MIND inherently assign more weights to certain modalities? Could this be due to intrinsic differences in data quality, signal-to-noise ratio, or the inherent discriminative power of each modality? A discussion on this point is crucial for understanding the method's integration priorities and potential biases.

6. In the downstream classification tasks on the both synthetic and cancer datasets, could the authors clarify the reason for choosing XGBoost as the classifier? Did the authors perform experiments to observe how different hyperparameters affect classification performance? If different classifiers and hyperparameters are explored, introducing a validation set is necessary for a rigorous evaluation.

7. The experimental section primarily demonstrates overall results. I recommend adding ablation studies to quantify the contribution of the key innovation components. For example, by displaying the performance decline when the t-SNE-driven prior is removed.

8. The study fundamentally relies on neighborhood structure, estimated via t-SNE. This implicitly assumes that the neighborhood structure in each modality's raw feature space is reliable and biologically meaningful. This assumption should be discussed and justified more explicitly, or supported by sensitivity analysis of key t-SNE hyperparameters to demonstrate the robustness of the proposed method.

9. The Discussion section primarily summarizes the results without providing a broader perspective. I strongly recommend expanding it to include a dedicated paragraph that thoughtfully discusses the limitations of MIND and proposes clear, specific directions for future research.

Minor comments:
10. On Figure 3, there is an inconsistency in the color representation of the JASMINE method across subplots. Specifically, JASMINE is depicted in brown in subplots a, b, d, and e, but is shown in red in subplots c and f. To maintain visual consistency and avoid potential confusion for readers, it would be beneficial to use a consistent color for JASMINE throughout all panels of the figure.

11. On Page 8, for the Section "Prior distribution on embeddings", the methods for creating the "normalized and vectorized versions of D" must be explicitly stated. Specifically, the authors should define the normalization technique applied (e.g., row-wise, symmetric normalization, or others) and precisely what is meant by "vectorized" in this context.

12. On Page 9, line 6 of the third paragraph, there appears to be a typo in the sentence "Note that the prior we proposed in Sec does not factorise." The phrase "in Sec" is incomplete. Please check and correct this.

13. The method is referred to as "IntegrAO" in Figure 3 but appears as "IntegraO" in Table 1 and Table 3. Please verify the correct spelling and ensure it is consistent throughout the manuscript to avoid confusion.

(Remarks on code availability)
The provided GitHub repository looks good to me. It inludes all information needed to reproduce the results presented in the manuscript.

Reviewer #2

(Remarks to the Author)
The authors present MIND, a novel method for multimodal integration. MIND uses a VAE architecture to integrate the different modalities, using a modality-specific encoder and decoder, and an integration of the different modalities using the data's embeddings. MIND further uses affinity matrices as priors for the embeddings, and also a regularization term to make the modality-specific embeddings similar to each other. Importantly, MIND's architecture allows it to be used in scenarios where some samples don't have data measured in all modalities. The authors demonstrate MIND's performance on synthetic data, and on three different cancer datasets, on several computational tasks that include supervised and unsupervised learning.
The paper is interesting, clear and well written. The problem of multimodal integration of biological data is important and especially under-addressed in the context of datasets with missing data. Furthermore, the authors convincingly demonstrate that MIND performs well compared to other methods. However, the paper still requires more analyses to make it more complete, and suitable for publication in Nature Communications.
Major comments:
- The paper would greatly benefit from an analysis of MIND's robustness, runtime, and from ablation studies. Several analyses seem particularly important to this reviewer:
o How sensitive is MIND to the dimension of the embedding? The results the authors show for JASMINE and "augmented" JASMINE suggest VAEs might be sensitive to that parameter.
o How does the regularization term making the modality-specific embeddings closer to each other affect MIND's performance, especially in the context of missing data?
o How long does it take to train MIND, and to use it on new samples?
o Does MIND's performance improve thanks to its consideration of multimodal data? How does it compare to an architecture where all modalities are concatenated, and only a single encoder and decoder are used? (only relevant when there's no missing data)
Minor comments:
- All the real datasets the authors use are cancer datasets (patients or cell lines). The paper would benefit from showing MIND's utility in a context other than cancer.
- The authors use the "augmented" version of JASMINE only in Table 2. However, the rationale for augmenting JASMINE holds true for other analyses, and in Table 2 the augmented version is clearly better than JASMINE. The authors should use augmented JASMINE in other analyses too for comparison.
- For the TCGA and CCMA datasets, the authors use KNN to impute missing values. This could create an advantage for methods that use neighborhood information, like MIND. This reviewer is not particularly worried about this issue, because of MIND's good performance on the CCLE dataset, but MIND's performance would be more robustly shown if only features with full data are used (depending on how many such features are there in the data), or a different imputation method is used.
- The synthetic data, and especially its protein modality, are not challenging enough. Judging from Figure 2, the protein modality holds enough information to perform perfect clustering and classification. The synthetic data analysis would be more convincing if the noise is increased such that no single modality still holds all the relevant information for classification and clustering.
- The authors describe their use of affinity matrices in MIND, as well as their regularization making the embeddings similar for different modalities, but they highlight the former more than the latter. For example, the regularization is not mentioned in the abstract and in Figure 1. To this reviewer, the regularization seems essential for MIND, and especially for its support of missing data. The authors should at least add it to Figure 1.
- A couple of the display items could use additional explanations:
o In Figure 4, is each point a single feature in a single sample? Or is this some aggregation, such that each feature only appears once in the plot? The first option is probably correct, but this could be explicitly stated.
o The reconstruction accuracy for TCGA is strongly higher in Table 4 than in Table 2. This is probably because variance between cancers (Table 4) is higher than within cancers (Table 2), but this could use a brief explanation.
Typos and other comments:
- "… cross-modal regularisation to encourages consistency…" -> encourage
- "… affinity matrices on the penitent-level embeddings" -> patient-level
- "… with those of the VAE-based JASMINE,…" – this sentence implies that the authors only compare MIND to JASMINE, but they also compare to MOVE.
- "a embedding vector of length 66" -> an embedding
- "availabel" -> available

(Remarks on code availability)

Reviewer #3

(Remarks to the Author)
I co-reviewed this manuscript with one of the reviewers who provided the listed reports. This is part of the Nature

Communications initiative to facilitate training in peer review and to provide appropriate recognition for Early Career Researchers who co-review manuscripts.

(Remarks on code availability)
The authors have provided a documented code repository, which includes a comprehensive README with complete installation instructions and runnable examples for all datasets.

Version 1:

Reviewer comments:

Reviewer #1

(Remarks to the Author)
The authors have addressed the concerns raised in the first round of review. The newly added experiments and discussions, particularly the BRCA case analysis that elucidates some biological interpretability of the method, strengthen the manuscript. The inclusion of additional results figures and tables further substantiates their claims. Their effort to refine the work has resulted in meaningful improvements.

However, I have some further concerns and suggestions based on this revision.

1(a). The “barcode integration” proposed by the authors in this revision distinguishes itself at the problem level from “mosaic integration” by assuming that missingness is independent of batch. However, the MIND model seems to lack built-in mechanisms to remove batch effects that may exist in the data, even if they are weak or unstructured. As a result, the performance of MIND relies on the biological fidelity of the neighborhood structure in the raw data. A specific concern is that if batch effects are present in the raw data and significantly influence the neighborhood structure identified by t-SNE, this biased neighborhood information would potentially compromise the quality of the final embeddings.

1(b). In cancer multi-omics databases such as TCGA, is the assumption that “modality missingness in patients is independent of batch” entirely valid? Could sample processing, sequencing centers, etc., still introduce batch effects that are weakly correlated with missingness patterns? The authors are recommended to discuss the potential risks or supplement relevant analyses regarding the two concerns above.

2. There is an inconsistency between the subfigure labels and their corresponding descriptions in the captions of Fig. 2 and Fig. 3. The authors should carefully check these.

3. While the authors have diligently addressed each comment in the response letter, it would be beneficial for future revisions to explicitly indicate where changes have been made in the manuscript. For example, by providing specific pointers such as “Page X, Line Y” for each modification. This would greatly facilitate the review process and ensure all revisions are easily located.

(Remarks on code availability)
The authors have also improved their code, providing new example scripts used in the analyses.

Reviewer #2

(Remarks to the Author)
The authors have addressed most of our concerns in the updated version of the manuscript, which now demonstrates MIND’s robustness more convincingly. However, the issue of comparing MIND to the “augmented” version of JASMINE has not been satisfactorily addressed. If this issue is addressed, we view this work as suitable for publication in Nature Communications.
The authors state that comparing MIND’s 64-dimensional representation to JASMINE with a higher dimensional (e.g 384) representation is advantageous for JASMINE. But the fact that MIND uses 64 dimensions does not necessarily mean that JASMINE should be used with 64 dimensions – it should be used with however many dimensions its authors recommended it to be used. If 64 is the dimension that the authors recommended for JASMINE then the current analysis is valid, but if not, making the comparison to JASMINE with 384 dimensions would show MIND’s merit in a more convincing manner.
Other minor comments for the manuscript:
- In Tables 3 and 4 of the rebuttal, it is not clear what is MIND_t, MIND_r and MIND_VAE. The authors should briefly explain this, potentially in the table legends.
- In the analysis using CCLE comparing single-omic to multi-omic performance, the authors should compare the performance of each single-omic to the same set of patients using the multi-omic data. While we understand that the fact that more patients can be used when doing the multi-omic analysis is an advantage of MIND, using the same set of patients (each time comparing one omic to multi-omic) is a more direct comparison. The authors can include both analyses.
- The authors state that they use solely cancer datasets due to data availability, but MIND would be more broadly applicable if the authors show that it can be used in multi-omic single-cell data, which are already available and are further increasing in availability (e.g. single-cell RNA + single-cell ATAC).
- The updated schematic figure is clearer, but it still does not show that MIND encourages the h representations for the different omics to be similar to each other, which we think is a key part of the algorithm.
- Typo: “to align different data modality to…” should be modalities.

(Remarks on code availability)

Reviewer #3

(Remarks to the Author)
I co-reviewed this manuscript with one of the reviewers who provided the listed reports. This is part of the Nature Communications initiative to facilitate training in peer review and to provide appropriate recognition for Early Career Researchers who co-review manuscripts.

(Remarks on code availability)
The authors have also improved their code, providing new example scripts used in the analyses.

Version 2:

Reviewer comments:

Reviewer #1

(Remarks to the Author)
The authors have provided explanation regarding batch effects and the independence of modality missingness, and I am satisfied with the corresponding revisions made to the manuscript. But there is still a minor concern that the descriptions for Mosaic and Barcode integration in Figure 1 are not clearly distinguished from each other.

(Remarks on code availability)
I have reviewed the code over the past two review rounds.

Reviewer #2

(Remarks to the Author)
We thank the authors for their responses to our comments.
Just to clarify our comment regarding using MIND for single-cell data - we didn't mean to use MIND in any scenario of missingness. Rather, since MIND can be used for data without missingness as well, we meant that MIND can be applied to single-cell multi-omic data, such as data where both RNA and ATAC is measured from each single cell. While this would demonstrate MIND's applicability in additional scenarios, it can be left for future work.
We now believe this work is suitable for publication in Nature Communications.

(Remarks on code availability)

Reviewer #3

(Remarks to the Author)
I co-reviewed this manuscript with one of the reviewers who provided the listed reports. This is part of the Nature Communications initiative to facilitate training in peer review and to provide appropriate recognition for Early Career Researchers who co-review manuscripts.

(Remarks on code availability)

Open Access This Peer Review File is licensed under a Creative Commons Attribution 4.0 International License, which permits use, sharing, adaptation, distribution and reproduction in any medium or format, as long as you give appropriate credit to the original author(s) and the source, provide a link to the Creative Commons license, and indicate if changes were made.
In cases where reviewers are anonymous, credit should be given to 'Anonymous Referee' and the source.
The images or other third party material in this Peer Review File are included in the article's Creative Commons license, unless indicated otherwise in a credit line to the material. If material is not included in the article's Creative Commons license and your intended use is not permitted by statutory regulation or exceeds the permitted use, you will need to obtain permission directly from the copyright holder.
To view a copy of this license, visit https://creativecommons.org/licenses/by/4.0/

We thank the reviewers for their constructive and insightful comments. The following details our point-by-point response.

1 Reviewer #1

> 1. The field of multi-omics data integration is well-established, with numerous robust methods already in existence. While the technical performance of MIND is demonstrated, the manuscript currently has not sufficiently demonstrated its conceptual or practical novelty. To clearly delineate MIND's contribution, the authors should provide a more rigorous comparison with a wider set of established methods, such as MOFA (2018), MOGONET (2021), MIDAS (2024) and DLVPM (2025). Many of these methods, especially those designed for single-cell multi-omics (e.g., GLUE (2022), scMoMaT (2023), StabMap (2024)), are inherently capable of handling bulk omics data integration and already address challenges like data heterogeneity and missingness. It is necessary to describe the MIND's unique contribution more clearly in order to justify its place within the field of methodology.

We thank the reviewer for identifying the need to more clearly delineate our contribution. We have revised our Introduction and Figure 1 to illustrate the different data integration settings and the applicability of different methods to each and positioned MIND against these.

We now describe the "barcode integration" problem which MIND attempts to solve and describe how it differs from the "mosaic integration" problem common in single-cell omics. In mosaic integration, certain batches of cells will be associated with a certain subset of data modalities and there is an entanglement between batch and modality representation. The barcode integration problem is a version of mosaic integration where there is no correspondence between the modalities present for any object and batch, i.e. a patient may only have DNA and RNA measurements because those were the only measurements taken, not because they were in a batch of patients who all only had DNA and RNA taken.

Our work therefore most resembles latent variable models such as MOFA (Argelaguet et al., 2018), MOVE (Allesøe et al., 2023), and JASMINE (Ballard et al., 2025) which address the barcode integration by learning shared sample-level latent representations and marginalising over arbitrarily missing modalities, thereby avoiding the need for repeated modality overlap or batch-structured integration. MOFA achieves this with a linear factor model, while MOVE and JASMINE extend the framework using nonlinear VAE architectures with increasing flexibility. Conceptually, MIND differs from these existing multimodal VAEs by replacing the standard factorised latent prior with a data-driven, neighbourhood-preserving prior that regularizes the geometry of patient-level embeddings under arbitrary modality missingness.

2. In the both Abstract and Introduction sections, the manuscript claims that existing methods often rely on imputation or sample removal, which can lead to information loss, while MIND learns directly from incomplete data via a multimodal VAE. However, when pre-processing datasets such as TCGA and CCMA, the authors first remove features with missing rates exceeding 20%, and then apply KNN imputation to the remaining missing values. This results in the model being trained on an approximately complete matrix after imputation, rather than on the original complex missing data structure. This approach appears inconsistent with the motivation of this work. It is recommended that the authors retain the original missing data structure in real-data experiments or, at a minimum, include comparative experiments without KNN imputation.

Our data preprocessing steps (e.g. removing columns with missing rates exceeding 20% then applying KNN imputation) is adopted from Ma et al. (2025). We would like to clarify that for each modality, each row of the modality-specific data matrix corresponds to the data vector of a patient *present in this modality.* Hence for each modality, patients whose corresponding data are not available will not appear in the modality-specific data matrix. As a result, the removal and imputation steps are applied *only* to the missing entries in the data vectors of patients *already present* in a modality (i.e., if a patient is present in a modality and the corresponding data vector contains missing values, the preprocessing steps will impute those missing values using KNN. However, if a patient is not present in a modality at all, its corresponding data remain completely missing).

MIND specifically focuses on addressing "missing patients in a modality" rather than "missing features in a patient's data vector". Let N be the total number of unique patients in the multiomics dataset, and l_m be the feature dimension of modality m. One could interpret the imputed data of modality m as a $N \times l_m$ matrix where the ith row is completely missing if the ith patient is not present in modality m. As a result, after the removal and imputation steps described above, the resulting data still has complex missing data structure, and the model is not being trained on a nearly complete dataset. We have highlighted this difference in the revised text (Data preprocessing section).

3. In the second paragraph of the Introduction section, the authors states: "This design principle enables the model to maintain interpretable and biologically meaningful representations in biomedical domains, while also generalising to any multimodal learning setting where incomplete data and heterogeneous noise are pervasive." This is a valuable claim. However, the Results section currently lacks a dedicated analysis to substantiate it. To fully support this statement and enhance the impact of the work, the authors are encouraged to include a specific case study that explicitly demonstrates how the learned representations are interpretable and lead to biologically meaningful insights. For instance, analyzing what the model has learned about specific genes or pathways would be highly compelling.

4. The manuscript evaluates MIND's performance on supervised and unsupervised tasks such as survival prediction, cancer type classification, and data reconstruction. However, a significant weakness is the exclusive reliance on quantitative metrics, failing to leverage these results to deliver new biological insights. The authors claim that "MIND can better extract biologically meaningful information from incomplete multi-omics data." However, this claim remains unsubstantiated by any concrete biological interpretation from the results. It is highly recommended that the authors provide analysis addressing the following questions: a) How do the learned embeddings or the integrated view from MIND help us understand the biological processes in the studied datasets (TCGA, CCMA, and CCLE)? An analysis linking the model's feature representations to established biology is crucial for interpretability. b) Can MIND generate novel biological hypotheses? The authors should present some specific case studies derived from their analysis. For example, did MIND identify a patient subgroup with distinct molecular features, a corresponding survival difference that might be clinically relevant, or a biologically meaningful regulatory network that was missed by other methods? Can this model accurately identify the biomarkers that contribute most significantly to cancer types?

We thank the reviewer for their suggestion. We address Point 3 and 4 here. In this revision, we included an examination the biological interpretability of the embeddings using the TCGA BRCA dataset as a representative case study. MIND achieved the strongest separation in survival risk for a four-cluster configuration (Figure 1a and Supplementary Figure S1) which highlighted distinct low- to high-risk survival profiles (Figure 1c). (See also Supplementary for details.)

We compared the MIND clusters to the PAM50 molecular subtypes assignments for these tumours (Figure 1b). We observed that some PAM50 `BRCA-LumA` and `BRCA-Normal` tumours were grouped into a single MIND cluster (No. 4). Prior work has shown that the `BRCA-Normal` subtype likely reflects `BRCA-LumA` tumours with low tumour purity (The Cancer Genome Atlas Network, 2012; Aran et al., 2015), with subsequent analyses reporting that 67.5% of `BRCA-Normal` tumours are reclassified as `BRCA-LumA` following appropriate adjustment (Veerla et al., 2024). MIND cluster No. 2 saw merging of `BRCA-LumB` and `BRCA-Her2`, which have been shown to converge on a shared proliferation-associated gene expression programme (Sotiriou et al., 2006), and approximately 30% of clinically `BRCA-Her2` tumours exhibit a `BRCA-LumB` intrinsic profile Prat et al. (2014). MIND cluster No. 1 corresponds to a basal subtype. We report the number of patients within each cluster identified by MIND and each PAM50 molecular subtype in Table 1.

MIND cluster No. 3 identified a distinct group of patients, predominantly of subtype `BRCA-LumA`, which exhibited markedly lower survival probabilities, suggesting a potential high-risk subgroup within `BRCA-LumA` (Figure 1c). Guo et al. (2019) identified a high-risk, Luminal-A–dominant breast cancer subtype (C3) with distinct inflammatory characteristics that exhibits elevated expression of granulocyte colony-stimulating factor (G-CSF) accompanied by neutrophil aggregation. To assess whether Cluster No.3 identified by MIND corresponds to this subtype, we examined CSF3 mRNA expression as a proxy for the high G-CSF phenotype. Specifically, we compared CSF3 expression levels between `BRCA-LumA` patients in Cluster No.3 (`LumA-High`) and those in Cluster No.4 (`LumA-Low`). As shown in Figure 1d, `LumA-High` patients exhibited substantially higher CSF3 expression, consistent with the defining characteristic of the C3 subtype. Figure 1f further showed that patients in Cluster No.3 show higher CSF3 expression than the other clusters.

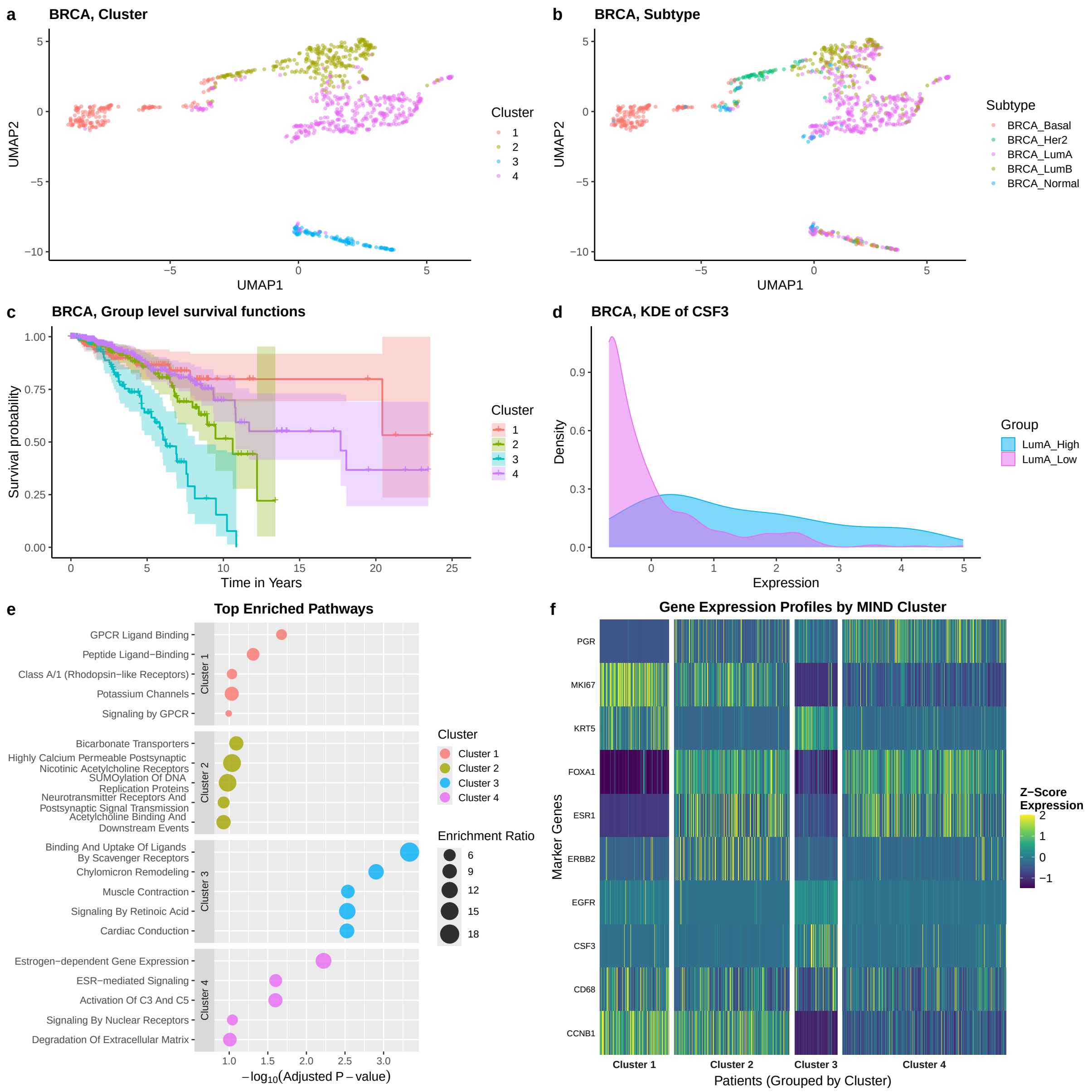

Figure 1: Clustering of BRCA: (a) UMAP of embeddings learnt by MIND, colored by clustering configuration identified by K-means with $K = 4$. (b) UMAP of embeddings learnt by MIND, coloured by cancer subtypes. (c) Estimated group level survival curve for each cluster in (a). (d): KDE of expression level of CSF3 for `BRCA-LumA` patients belonging to Cluster 3 and 4 in (a) respectively. (e) Top five enriched pathways with $-\log_{10}(p_{adj})$ and enrichment ratios for each of the clusters identified by MIND. (f) Heat map of standardised mRNA expressions of a subset of marker genes of patients in BRCA dataset.

To characterise the biological relevance of MIND-derived patient clusters, we further performed a gene set enrichment analysis: We first identified differentially expressed genes (DEGs) for each cluster by performing a one-vs-rest comparison using a two-sided t-test on the standardised mRNA expression values of the top 2,000 most variable genes. Genes were considered potential markers if they exhibited a positive t-statistic. We then ranked these genes by their test statistic and performed an over-representation analysis using the Reactome 2022 pathway

database (Gillespie et al., 2022) via the Enrichr server (Xie et al., 2021) through `gseapy` library. Pathways with an adjusted p-value less than 0.05 were considered statistically significant. Top 5 enriched pathways for each cluster are reported in Table 2 and Figure 1e.

We validated the biological relevance of MIND-derived clusters by examining their enriched molecular pathways. We found that Cluster No. 4 showed significant enrichment for Estrogen-dependent Gene Expression (p_{adj}=0.006) and ESR-mediated signalling (p_{adj}=0.025). These findings are consistent with the clinical profile of Luminal A breast cancer (Miah et al., 2019; Yersal and Barutca, 2014), confirming that MIND correctly identifies the hormone-driven Luminal A phenotype which is associated with better survival outcomes. In contrast, Cluster No. 3, which we hypothesised corresponded to the high risk, inflammatory C3 subtype, showed significant enrichment for Scavenger Receptor Ligand Binding and Uptake ($p_{adj} < 0.001$). Scavenger receptors are predominantly expressed in macrophages and other innate immune cells (Peiser et al., 2002), suggesting that Cluster No. 3 is also characterised by distinct immune infiltration and inflammatory signalling rather than pure hormonal drivers. This further supports the correspondence between Cluster No. 3 and inflammatory C3 subtype. Additionally, Cluster No. 1, which corresponded to the Basal subtype, showed enrichment for GPCR Ligand Binding (p_{adj} = 0.021), a pathway frequently implicated in the aggressive metastatic potential of basal and triple-negative breast cancers (Feigin et al., 2014), contrasting with the hormone-driven pathways of Luminal tumours. This further confirms the biological relevance of MIND embeddings.

Together, these analyses indicate that MIND-derived embeddings recover known biological structure and support the identification of clinically and biologically interpretable patient subgroups.

> 5. The results in Figure 2 raises an important question regarding the integration behavior of MIND. It is evident that the structures in the individual modality UMAPs (a-c) vary in clarity, with Protein modality (Fig.2a) exhibiting the most distinct separation, while the DNA methylation modality (Fig.2c) appears to contribute less to the final result. The authors should discuss the potential reasons for this observation. For instance, does MIND inherently assign more weights to certain modalities? Could this be due to intrinsic differences in data quality, signal-to-noise ratio, or the inherent discriminative power of each modality? A discussion on this point is crucial for understanding the method's integration priorities and potential biases.

The data used for the synthetic data example (Fig 2) is generated using the `InterSim` CRAN package (Chalise et al., 2016). The different levels of separation shown in the the individual modality UMAPs are controlled by the different input parameters used to simulate the synthetic datasets from each of the three modalities. The different levels of cluster separation on the synthetic data are deliberately introduced (by using different simulating parameters) to, as the reviewer pointed out, mimic real world multiomics datasets whose data quality, signal-to-noise ratio and/or inherent discriminative power may vary across different modalities.

To further examine how the data quality and/or signal-to-noise ratio of the observed data affect integration performance, we added a more noisy scenario in which synthetic data from all three modalities show no clear cut clustering structures. We see that in this case, MIND achieved similar performance to JASMINE, and outperformed MOVE, IntegrAO and MSNE in terms of classification and clustering accuracy, and outperformed all methods in terms of reconstruction accuracy. (Fig 2, 3). Details can be found in the Synthetic data section of the

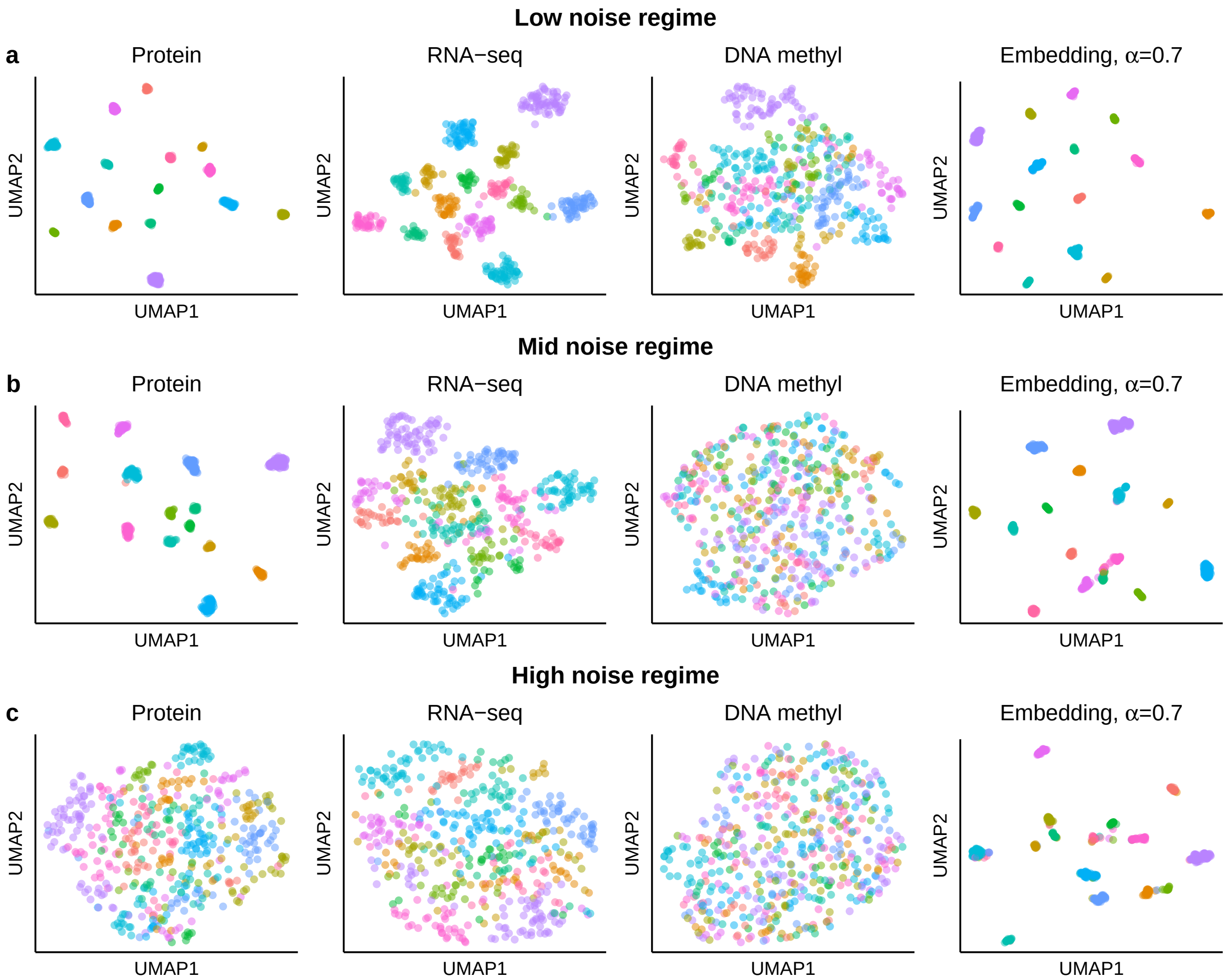

Figure 2: Visualisation of synthetic datasets and embeddings. Top row: Low noise regime. (a-c) UMAP visualisation of the three modalities. (d). UMAP visualisation of the embeddings estimated by the proposed method at overlap ratio $\alpha = 0.7$. Mid row: Similar figures under the mid noise regime. Bottom row: Similar figures under the high noise regime.

revised manuscript. Relevant datasets have been uploaded to Figshare.

Our proposed MIND treat neighbourhood information from each modality equally. To see that, note the contribution of the KL terms in the exponentially tilted prior (Eqn 2 in main text) from all modalities are equal. As a result, MIND would not inherently favour certain modality. We agree with the reviewer that allowing MIND to adaptively assigning different weights to different modalities by e.g. dynamically reweighting the contribution of the neighbourhood structures from different modalities can help MIND filter out non-informative modalities and focus on the informative ones, and would be a very promising future direction for improving robustness of MIND. We have added the discussion to the Discussion section of the revised manuscript.

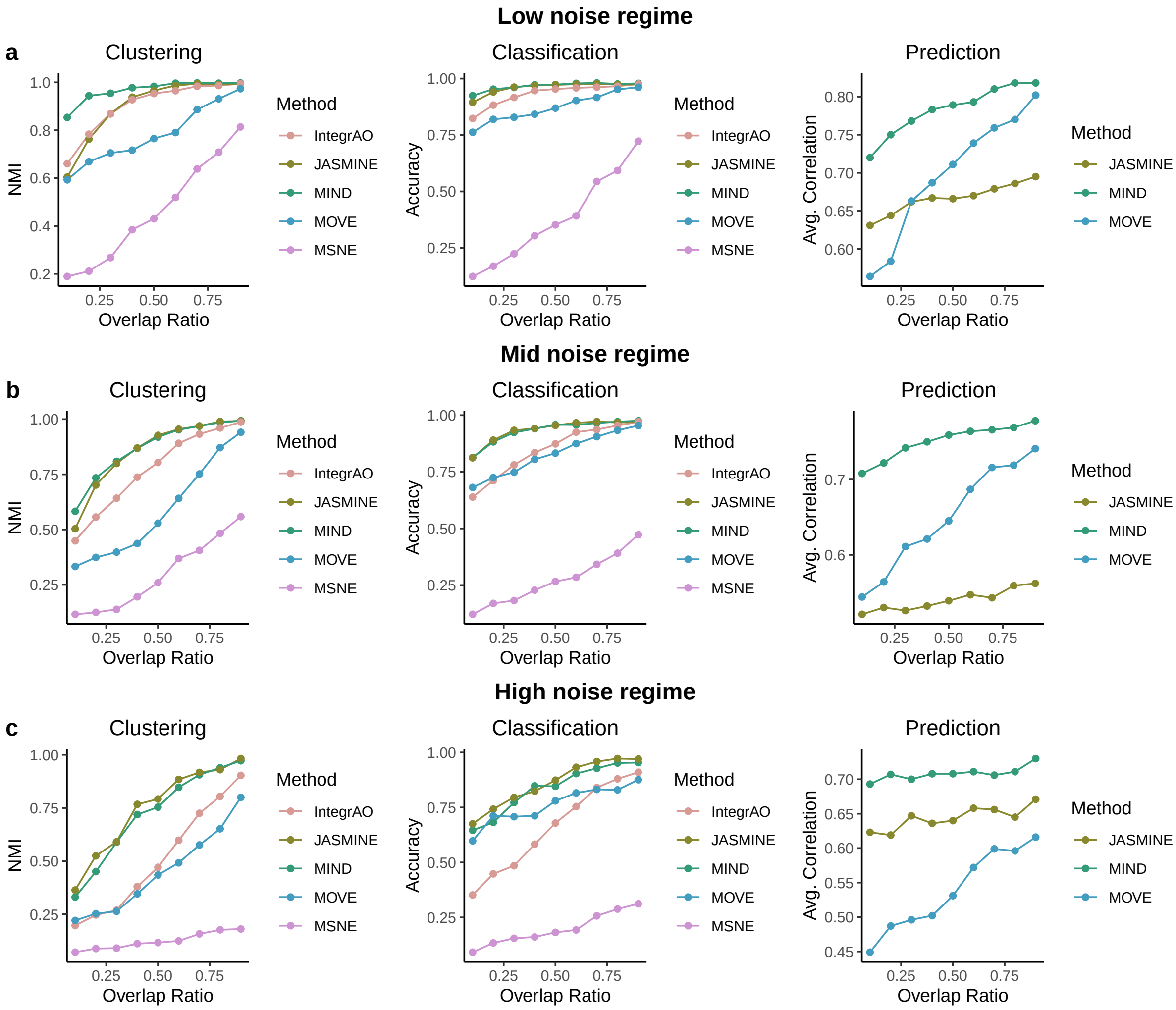

Figure 3: Performance on the three downstream tasks. Top row: Results from the low noise datasets. (a) Normalised Mutual Information vs overlapping data ratio of different methods. (b) Classification accuracy estimated by 10-fold CV of different methods. (c) Prediction accuracy measured by the Pearson correlations between the masked portion of the synthetic data and the predicted, averaged over all modalities. Mid row: Similar results from the mid noise datasets. Bottom row: Similar results from the high noise datasets.

> 6. In the downstream classification tasks on the both synthetic and cancer datasets, could the authors clarify the reason for choosing XGBoost as the classifier? Did the authors perform experiments to observe how different hyperparameters affect classification performance? If different classifiers and hyperparameters are explored, introducing a validation set is necessary for a rigorous evaluation.

We chose to use XGBoost with default settings *without* finetuning the hyperparamters. In the benchmarking section, we compare the quality of embeddings generated by different integration methods by using them to train the *same* XGBoost classifier model and then investigating their performances. We did not experiment with different hyperparamters because we wanted to use the "default classifier" as a fair and robust test ground, as tuning hyperparmeters based on the performance of any of the data integration methods would be unfair to the rest. Indeed, finetuning the classifier hyperparamters for embeddings generated by different integration methods individually would lead to better performances for each of the methods including MIND, but we believe comparing the best each integration method can achieve is less meaningful than comparing their performances using the same "baseline" classifier. For example, suppose two sets of embeddings $\mathbf{U}, \mathbf{V}$ learnt by two integration methods on the same dataset achieve the same classification accuracy with different choices of hyperparameters (e.g. number of trees N_{tree} in XGBoost). If the model trained on $\mathbf{U}$ has a much simpler structure (e.g. a small N_{tree}) than the one trained on $\mathbf{V}$ (e.g. vert large N_{tree}), then $\mathbf{U}$ should still be favourable for sake of parsimony. In our example, the "default" XGBoost classifier can be viewed as a fixed "complexity baseline" for integration methods with e.g. potentially different latent space geometries.

In this paper, XGBoost classifier was chosen because of its robustness and efficiency, but we agree with the reviewers that XGBoost is just one of the many classifiers that could be used for benchmarking downstreaming task performances. We therefore follow Ma et al. (2025), and included similar benchmarking results using classifiers including Neural network, Support Vector Machine, Random Forest, and K-Nearest Neighbour in additional to XGBoost. Results are reported in Table 9, 10, 11. We halve also added the results to the revised text. Relevant scripts have been uploaded to Github

> 7. The experimental section primarily demonstrates overall results. I recommend adding ablation studies to quantify the contribution of the key innovation components. For example, by displaying the performance decline when the t-SNE-driven prior is removed.

We have added an ablation study to investigate the contribution of both the t-SNE prior and the patient level regularisation using the CCLE dataset as an example. Specifically, we fix embedding size to be 64 and perplexity parameter to be 30, and fit four variants of MIND: MIND_{VAE} (without either the t-SNE informed prior or the patient level regularisation), MIND_t (with the t-SNE informed prior but without the patient level regularisation), MIND_r (without the t-SNE informed prior but with the patient level regularisation, $\alpha = 0.05$), and MIND (with both the t-SNE informed prior and the patient level regularisation, $\alpha = 0.05$). (We put isotropic Gaussian prior on the embeddings whenever the t-SNE informed prior is not used.) We found that the inclusion of both the t-SNE informed prior and the patient-level regularisation improves the performance of MIND in both classification and reconstruction accuracy, confirming the efficacy of both terms (Table 3, 4).

We have also investigated the impact of the perplexity parameter of t-SNE by fixing the em-

bedding size at 64, and comparing the performance of MIND with perplexity parameter set to $8, 15, 30, 45, 60$. We found MIND is robust under various choices of t-SNE perplexity parameters (Table 5, 6).

Finally, we investigate the impact of embedding sizes. Here we fix the perplexity parameter at 30, and experiment with embedding size $8, 16, 32, 64, 128, 256$. We found that increasing embedding size leads to a diminishing improvement in performance of downstreaming tasks such as classification accuracy, but also a poorer performance on reconstruction accuracy (Table 7, 8). This is not surprising as higher dimensional embeddings have the additional flexibility to capture finer details of the dataset. But this flexibility also makes the model prone to e.g. overtraining that leads to the poor performance on data reconstruction. As a result, we recommend setting a moderate embedding size $d = 64$ to provide sufficient flexibility while preventing over-fitting. Results above are added to the Ablation studies of the Supplementary Material.

> 8. The study fundamentally relies on neighborhood structure, estimated via t-SNE. This implicitly assumes that the neighborhood structure in each modality's raw feature space is reliable and biologically meaningful. This assumption should be discussed and justified more explicitly, or supported by sensitivity analysis of key t-SNE hyperparameters to demonstrate the robustness of the proposed method.

We agree with the reviewer that MIND relies on the assumption that local manifold structure (i.e., nearest neighbourhood relationships) learnt from raw multi-omics data preserves significant biological signals. This neighbourhood structure learnt from raw data is not perfect since it can be contaminated by e.g. inherent noise and technical artifacts. The synergy between the t-SNE-driven prior and the Multimodal VAE architecture in MIND is specifically designed to mitigate this issue: The exponentially tilted prior incorporates (imperfect) neighbourhood structure from the raw data, and guides the latent space to respect that local topology of the patients, preventing the VAE from learning purely reconstruction-guided embeddings that ignores biological similarity, while the VAE component prevents the embeddings from simply memorizing the noisy t-SNE projections if those projections contradict the underlying feature patterns learnt from reconstruction. Therefore the t-SNE-informed prior does not dictate the result alone. Instead, it acts as a topological reference point (i.e. a soft constraint), while the VAE allows the model to deviate from the imperfect topological structure identified by t-SNE from the raw data where necessary to ensure reconstruction accuracy. Empirically, the ablation study also demonstrates that MIND is robust under difference choices of the perplexity parameter (Table 5, 6). We have added the discussion to the Method section of the revised text.

> 9. The Discussion section primarily summarizes the results without providing a broader perspective. I strongly recommend expanding it to include a dedicated paragraph that thoughtfully discusses the limitations of MIND and proposes clear, specific directions for future research.

While MIND demonstrates robust performance in integrating incomplete multi-omics data, several limitations remain. First, although MIND effectively addresses the challenge of "missing patients in a modality" (where an entire data matrix row is absent), it currently relies on preprocessing steps, such as KNN imputation, to handle "missing values within a data vector". This two-step approach creates a disconnect between the imputation of feature-level missingness and the learning of patient-level embeddings. A valuable direction for future work would be to

extend the probabilistic framework of MIND to natively handle both types of missingness simultaneously via e.g. multiple imputation (Rubin, 2018). By modelling missing features within the VAE architecture itself, we could eliminate the reliance on external imputation methods and potentially improve the preservation of biological signals . Second, the current formulation of MIND treats the neighbourhood information derived from different modalities equally through the exponentially tilted prior. However, multi-omics datasets often exhibit varying levels of signal-to-noise ratios and discriminative power across modalities (Table 15). In scenarios where specific modalities are dominated by technical noise or batch effects, equal weighting may be suboptimal. Future iterations of MIND could incorporate adaptive weighting mechanisms, such as dynamic reweighting or attention modules, to automatically up-weight informative modalities and down-weight noisy ones. This would further enhance the model's robustness against heterogeneous noise sources inherent in complex biological assays. Finally, MIND is currently designed as a "static" integration method, optimized for learning informative embeddings from a specific patient cohort rather than predicting missing modalities for completely new, out-of-sample patients. Direct application of the trained model to new samples carries the risk of performance degradation due to distribution shifts between the training cohort and the new data. To address this, a promising future direction is the development of a dedicated prediction module capable of explicitly modelling and correcting for distribution shifts. Such an extension would enable statistically principled predictions for new patients, significantly broadening the clinical applicability of the framework for tasks such as precision medicine and diagnostic classification. We have added the discussion above to the Discussion section of the revised text.

> 10. On Figure 3, there is an inconsistency in the color representation of the JASMINE method across subplots. Specifically, JASMINE is depicted in brown in subplots a, b, d, and e, but is shown in red in subplots c and f. To maintain visual consistency and avoid potential confusion for readers, it would be beneficial to use a consistent color for JASMINE throughout all panels of the figure.

We have updated the figures with consistent coloring (Fig 3)

> 11. On Page 8, for the Section "Prior distribution on embeddings", the methods for creating the "normalized and vectorized versions of D" must be explicitly stated. Specifically, the authors should define the normalization technique applied (e.g., row-wise, symmetric normalization, or others) and precisely what is meant by "vectorized" in this context.

The normalised and vectorised $\bar{\mathcal{D}}^m$ matrices are obtained by following the standard t-SNE computation pipeline: an affinity matrix $\mathcal{D}^m$ is first normalised by dividing each entry in $\mathcal{D}^m$ by the sum of all entries of $\mathcal{D}^m$, then flattened into a $|B^m|^2 \times 1$ vector by stacking all columns of the normalised matrix. This operation turns an affinity matrix $\mathcal{D}^m$ into a probability vector that sums to 1. We have added the details into the Method section of the revised text.

> 12. On Page 9, line 6 of the third paragraph, there appears to be a typo in the sentence "Note that the prior we proposed in Sec does not factorise." The phrase "in Sec" is incomplete. Please check and correct this.

Typos have been corrected in the revised text.

13. The method is referred to as "IntegrAO" in Figure 3 but appears as "IntegrAO" in Table 1 and Table 3. Please verify the correct spelling and ensure it is consistent throughout the manuscript to avoid confusion.

Typos have been corrected in the revised text.

2 Reviewer #2

1. The paper would greatly benefit from an analysis of MIND's robustness, runtime, and from ablation studies. Several analyses seem particularly important to this reviewer:

- How sensitive is MIND to the dimension of the embedding? The results the authors show for JASMINE and "augmented" JASMINE suggest VAEs might be sensitive to that parameter.
- How does the regularization term making the modality-specific embeddings closer to each other affect MIND's performance, especially in the context of missing data?
- How long does it take to train MIND, and to use it on new samples?
- Does MIND's performance improve thanks to its consideration of multimodal data? How does it compare to an architecture where all modalities are concatenated, and only a single encoder and decoder are used? (only relevant when there's no missing data)

- We have added an ablation study using the CCLE dataset to the Supplementary materials. We found that increasing embedding size leads to a diminishing improvement in performance of downstreaming tasks such as classification accuracy, but also a poorer performance on reconstruction accuracy (Table 7, 8). This is not surprising as higher dimensional embeddings have the additional flexibility to capture finer details of the dataset. But this flexibility also makes the model prone to e.g. overtraining that leads to the poor performance on data reconstruction. As a result, we recommend setting a moderate embedding size (e.g. $d = 64$) to provide sufficient flexibility while preventing over-fitting. Relevant scripts have been uploaded to Github

- We have also performed an ablation study to investigate the impact of the regularisation term and the exponential tilting term in the t-SNE informed prior. We found the inclusion of this regularisation improves the performance of MIND, and synergises well with the t-SNE informed prior (Table 3, 4). Results have also been added to the Supplementary material.

- Running times of all methods are reported in Table 14. All running times are record on our desktop with an AMD 5700 CPU and a Nvidia RTX2060 GPU. We found that network-based methods (IntegrAO and MSNE) are faster to train. Among VAE based methods, MIND and MOVE have similar training time, while the training time for JASMINE is longer, which is likely due to its additional cross modality modules. Results have been added to the revised text.

  Regrading predicting new samples, we recommend not to use MIND on new sample prediction because of the risk of distribution shift: In practice, a new patient's multi-omics data does not necessarily follow the same distributions of the data of the patient cohort used to train MIND. This discrepancy between the "training" and "query" data distributions (i.e. distribution shift) will likely lead to inaccurate prediction and potentially misleading

clinical decisions for the new patient. MIND is designed to learn a set of informative patient level embeddings by integrating an existing, "static" incomplete multiomics dataset, rather than predicting the missing data of a new patient given the new patient's available modalities and a previously observed "reference" dataset. We agree with the reviewer that an integration method with the ability to predict missing modality of a new sample will be highly beneficial. One potential extension of MIND is to incorporate such prediction module that is robust against distribution shift in a statistically principled way. We have added the paragraph to the Discussion section of the revised text.

- We use CCLE dataset as an example to illustrate how integrating multimodal data improves MIND's performance on cancer type classification. We compare the performance of cancer type classification accuracies of MIND embeddings estimated based on the multimodal dataset and each of the individual datasets (RNA, DNA methylation, CNV, miRNA, RPPA, and Metabolomics). We found the accuracy of the multimodal MIND is consistently higher than those trained on individual datasets, confirming that integrating multimodal data improves the performance of the learnt embeddings on downstreaming tasks (Table 15). Note that for embeddings trained on individual datasets, the classification accuracy are estimated based on all the patients present in that modality. In contrast, for embeddings trained on multimodal dataset, the classification accuracy is estimated from all available patients in the cohort. As a result, incorporating multimodal data also allows us to analyse a larger set of patients using a single representation learning model. We have added the results to the revised main text.

  If all data are available, we expect the performance would be better than the current model based on incomplete data because of the additional information. However, under the presence of complex missing pattern, developing statistically and computationally efficient ways to handle missing modalities by concatenating available features can be very challenging. This is exactly the point of multimodal integration methods including MIND: They aim to integrate all available data when complex missing patterns make training a single VAE model by concatenating features infeasible or statistically inefficient.

> 2. All the real datasets the authors use are cancer datasets (patients or cell lines). The paper would benefit from showing MIND's utility in a context other than cancer

We acknowledge the reviewer's concern regarding the focus on cancer datasets. While the current evaluation relies on cancer cohorts such as TCGA and CCMA, this choice is primarily driven by the availability of high-quality, publicly available multi-omics data with well-defined clinical outcomes. In contrast, multi-omics datasets for other health conditions are often subject to usage restrictions. For instance, the Alzheimer's dataset used by JASMINE (Ballard et al., 2025) is not publicly available. Furthermore, large-scale cohort studies such as the UK Biobank frequently rely on biological samples collected at baseline during recruitment, which may have a relatively weak or distal relationship to future disease states compared to the more immediate molecular signatures found in cancer biopsies. Conceptually, however, there is no inherent reason why MIND's architecture, designed to handle heterogeneous noise and complex missing patterns, would be invalid for other therapeutic or clinical areas, and exploring its utility in diverse health conditions remains a priority for our future research. We have added the discussion to the Discussion section of the revised manuscript.

3. The authors use the "augmented" version of JASMINE only in Table 2. However, the rationale for augmenting JASMINE holds true for other analyses, and in Table 2 the augmented version is clearly better than JASMINE. The authors should use augmented JASMINE in other analyses too for comparison.

The patient embeddings of JASIMNE are generated by concatenating modality-level embeddings learned from modality-specific sub VAEs. As a result, to get a e.g. $d = 64$ dimensional final embedding in the real world examples, the modality-level embeddings produced by modality-specific sub VAEs can only have length 10 to 12. Since JASMINE reconstructs data from different modalities by passing the modality-level embeddings of size 10 to 12 to modality-specific sub decoders, we hypothesise that these very low dimensional modality-level embeddings might be too stringent for the sub decoders to excel in data reconstruction. As a result, we additionally incorporate the augmented JASMINE to further test its reconstruction performance when individual modality-level embeddings are allowed to have the same size as the full patient level embedding of MIND. This is a very advantageous scenario for JASMINE. On the other hand, for downstreaming tasks that aim to compare the quality of embeddings and do not require modality-specific sub decoders (e.g. classification, clustering, survival prediction), we do not consider the augmented JASMINE because the length of its final embedding is 4 to 6 times longer than outputs of the rest of the methods. For example, for the TCGA dataset, when we set the embedding size $d = 64$, the augmented JASMINE would output embeddings of length 384. We think it is not meaningful to directly compare the $d = 64$ dimensional embeddings generated by MIND and IntegrAO with 384 dimensional embeddings given by the augmented JASMINE. We therefore only compare JASMINE with the rest of the methods at the same embedding size for all tasks except for reconstruction.

4. For the TCGA and CCMA datasets, the authors use KNN to impute missing values. This could create an advantage for methods that use neighborhood information, like MIND. This reviewer is not particularly worried about this issue, because of MIND's good performance on the CCLE dataset, but MIND's performance would be more robustly shown if only features with full data are used (depending on how many such features are there in the data), or a different imputation method is used.

We use the CCMA dataset as an example to illustrate the impact of imputation. We benchmark different models on a trimmed version of CCMA such that only features with full data are included (i.e. all columns containing missing values are removed) in a similar fashion to the imputed case. Results are reported in Table 12, 13. Similar to the imputed case, MIND demonstrated the best performance. We also found that cancer type classification accuracies of all methods were worse than ones based on the imputed data. This is likely due to the fact that informative features were removed in the trimming process. On the other hand, the reconstruction performances were better. But we believe this is because the model is handling a easier reconstruction task (a much smaller dataset), not because it has learnt the underlying biological signal better. We have added the results to the revised Supplementary material.

5. The synthetic data, and especially its protein modality, are not challenging enough. Judging from Figure 2, the protein modality holds enough information to perform perfect clustering and classification. The synthetic data analysis would be more convincing if the noise is increased such that no single modality still holds all the relevant information for classification and clustering.

To further examine how the data quality and/or signal-to-noise ratio of the observed data affect integration performance, we added a more challenging scenario where synthetic data from all three modalities show no clear cut clustering structures. We see that in this case, MIND achieved similar performance to JASMINE, and outperformed MOVE, IntegrAO and MSNE in terms of classification and clustering accuracy, and outperformed all methods in terms of reconstruction accuracy. (Fig 2, 3). We have added the results to the revised manuscript. Relevant datasets have been uploaded to Figshare.

6. The authors describe their use of affinity matrices in MIND, as well as their regularization making the embeddings similar for different modalities, but they highlight the former more than the latter. For example, the regularization is not mentioned in the abstract and in Figure 1. To this reviewer, the regularization seems essential for MIND, and especially for its support of missing data. The authors should at least add it to Figure 1.

We have updated the illustration figure (Fig 4). The patient level regularisation step has been added to the illustration with a brief introduction.

7. A couple of the display items could use additional explanations:
- In Figure 4, is each point a single feature in a single sample? Or is this some aggregation, such that each feature only appears once in the plot? The first option is probably correct, but this could be explicitly stated.
- The reconstruction accuracy for TCGA is strongly higher in Table 4 than in Table 2. This is probably because variance between cancers (Table 4) is higher than within cancers (Table 2), but this could use a brief explanation.

- In main text Fig 4, each point corresponds to one feature in a single sample. We have added the explanation to the caption.
- The better reconstruction accuracy of the full TCGA example compared to the individual ones is indeed due to the fact that the variances of data between individual cancer datasets are much larger than those within individual datasets We have added this point to the revised main text Fig 6.

Typos

Typos have been corrected in the revised text.

3 Reviewer #3

We thank the reviewer for supporting Early Career Researchers with peer review.

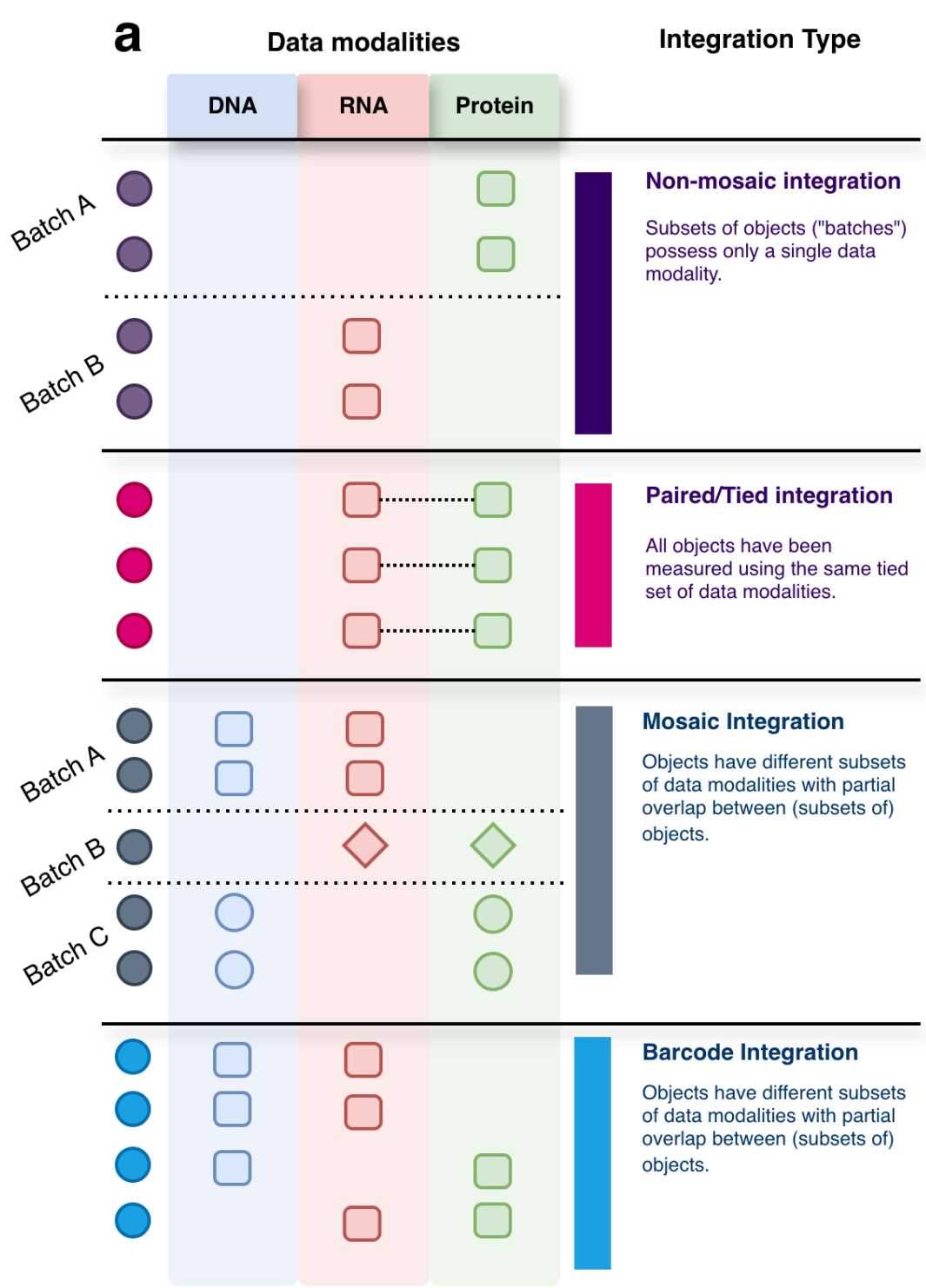

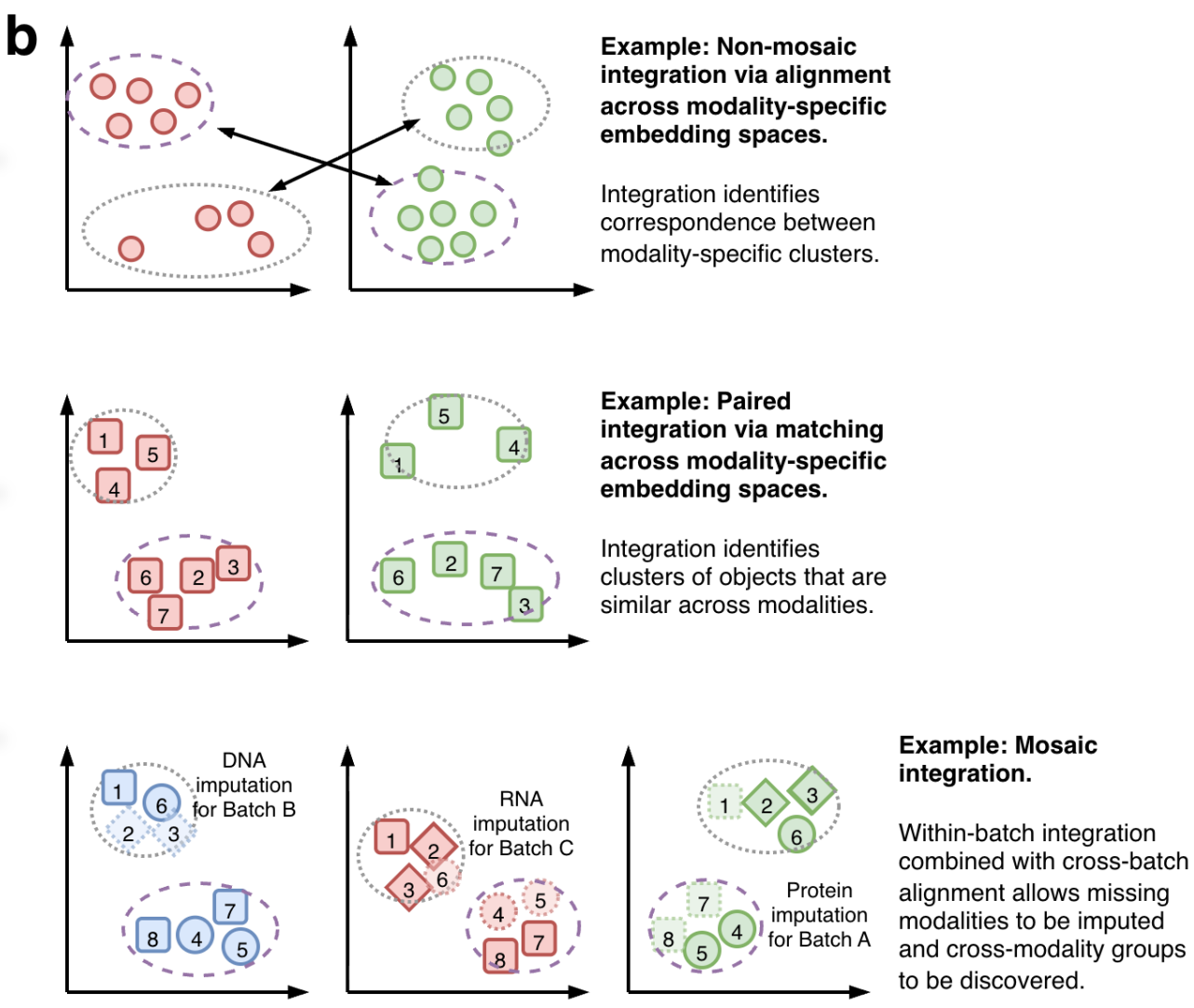

c

Method	Supervised	Single cell	Mosaic integration	Paired	Integrated embedding
MIND	N	N	N	Y	Y
MOFA	N	N	N	Y	Y
MOGONET	Y	N	N	Y	Y
DLVPM	N	N	N	Y	N
MIDAS	N	Y	Y	Y	Y
GLUE	N	Y	N	N	Y
scMoMaT	N	Y	Y	Y	Y
StabMap	N	Y	Y	Y	Y

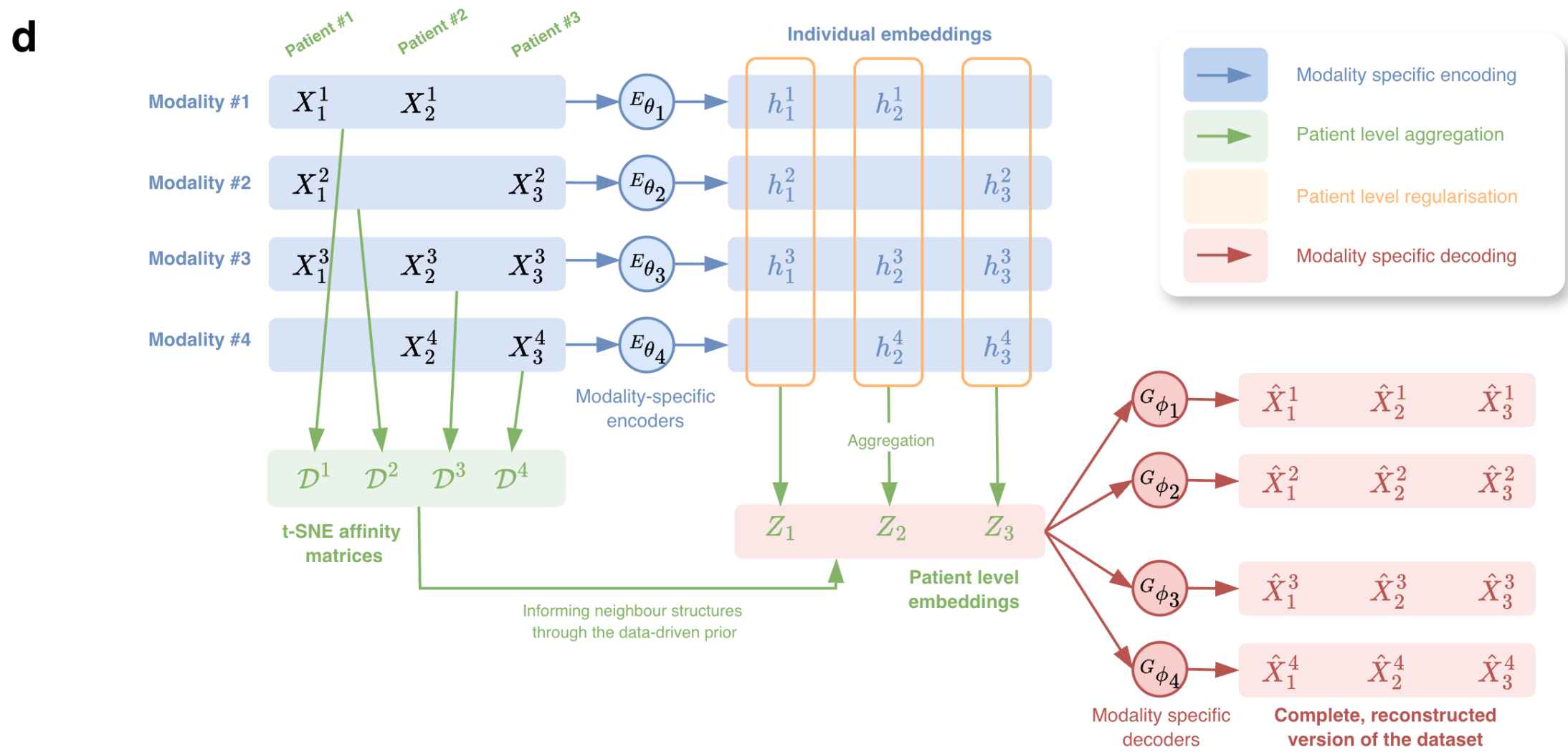

Figure 4: Schematic illustration of MIND. We use an incomplete multiomics dataset with $M = 4$ modalities and $N = 3$ patients as an example. Available X_n^ms in the incomplete dataset are first mapped to individual embeddings h_n^ms by modality-specific encoders $\{E_{\theta_m}\}_{m=1}^M$. Individual embeddings h_n^m associated with the same patient n are then aggregated into a patient level embedding Z_n with a patient-level regularisation on the corresponding h_n^ms. Each X_n^m is reconstructed (if available in the incomplete dataset) or predicted (if missing) by passing the patient level embedding Z_n to the modality-specific decoder G_{ϕ_m}. For each modality, a t-SNE affinity matrix $\mathcal{D}^m$ encoding neighbour structure of the relevant patients is computed. These matrices are used to guide the neighbour structure of patient level Z_ns through the prior on Z_ns.

References

Allesøe, R. L., Lundgaard, A. T., Hernández Medina, R., Aguayo-Orozco, A., Johansen, J., Nissen, J. N., Brorsson, C., Mazzoni, G., Niu, L., Biel, J. H., et al. (2023). Discovery of drug–omics associations in type 2 diabetes with generative deep-learning models. *Nature biotechnology*, 41(3):399–408.

Aran, D., Sirota, M., and Butte, A. J. (2015). Systematic pan-cancer analysis of tumour purity. *Nature communications*, 6(1):8971.

Argelaguet, R., Velten, B., Arnol, D., Dietrich, S., Zenz, T., Marioni, J. C., Buettner, F., Huber, W., and Stegle, O. (2018). Multi-omics factor analysis—a framework for unsupervised integration of multi-omics data sets. *Molecular systems biology*, 14(6):e8124.

Ballard, J. L., Dai, Z., Shen, L., and Long, Q. (2025). Jasmine: A powerful representation learning method for enhanced analysis of incomplete multi-omics data. *bioRxiv*, pages 2025–06.

Chalise, P., Raghavan, R., and Fridley, B. L. (2016). Intersim: Simulation tool for multiple integrative 'omic datasets'. *Computer methods and programs in biomedicine*, 128:69–74.

Feigin, M. E., Xue, B., Hammell, M. C., and Muthuswamy, S. K. (2014). G-protein–coupled receptor gpr161 is overexpressed in breast cancer and is a promoter of cell proliferation and invasion. *Proceedings of the National Academy of Sciences*, 111(11):4191–4196.

Gillespie, M., Jassal, B., Stephan, R., Milacic, M., Rothfels, K., Senff-Ribeiro, A., Griss, J., Sevilla, C., Matthews, L., Gong, C., et al. (2022). The reactome pathway knowledgebase 2022. *Nucleic acids research*, 50(D1):D687–D692.

Guo, L., Chen, G., Zhang, W., Zhou, L., Xiao, T., Di, X., Wang, Y., Feng, L., and Zhang, K. (2019). A high-risk luminal a dominant breast cancer subtype with increased mobility. *Breast Cancer Research and Treatment*, 175(2):459–472.

Ma, S., Zeng, A. G., Haibe-Kains, B., Goldenberg, A., Dick, J. E., and Wang, B. (2025). Moving towards genome-wide data integration for patient stratification with integrate any omics. *Nature Machine Intelligence*, 7(1):29–42.

Miah, S., Bagu, E., Goel, R., Ogunbolude, Y., Dai, C., Ward, A., Vizeacoumar, F. S., Davies, G., Vizeacoumar, F. J., Anderson, D., et al. (2019). Estrogen receptor signaling regulates the expression of the breast tumor kinase in breast cancer cells. *BMC cancer*, 19(1):78.

Peiser, L., Mukhopadhyay, S., and Gordon, S. (2002). Scavenger receptors in innate immunity. *Current opinion in immunology*, 14(1):123–128.

Prat, A., Carey, L. A., Adamo, B., Vidal, M., Tabernero, J., Cortés, J., Parker, J. S., Perou, C. M., and Baselga, J. (2014). Molecular features and survival outcomes of the intrinsic subtypes within her2-positive breast cancer. *Journal of the National Cancer Institute*, 106(8):dju152.

Rubin, D. B. (2018). Multiple imputation. In *Flexible imputation of missing data, second edition*, pages 29–62. Chapman and Hall/CRC.

Sotiriou, C., Wirapati, P., Loi, S., Harris, A., Fox, S., Smeds, J., Nordgren, H., Farmer, P., Praz, V., Haibe-Kains, B., et al. (2006). Gene expression profiling in breast cancer: understanding the molecular basis of histologic grade to improve prognosis. *Journal of the National Cancer Institute*, 98(4):262–272.

The Cancer Genome Atlas Network (2012). Comprehensive molecular portraits of human breast tumours. *Nature*, 490(7418):61–70.

Veerla, S., Kimbung, S., Ebbesson, A., Vallon-Christersson, J., and Hedenfalk, I. (2024). Deciphering the normal-like molecular subtype of breast cancer. *Cancer Research*, 84(3_Supplement_1):B054–B054.

Xie, Z., Bailey, A., Kuleshov, M. V., Clarke, D. J., Evangelista, J. E., Jenkins, S. L., Lachmann, A., Wojciechowicz, M. L., Kropiwnicki, E., Jagodnik, K. M., et al. (2021). Gene set knowledge discovery with enrichr. *Current protocols*, 1(3):e90.

Yersal, O. and Barutca, S. (2014). Biological subtypes of breast cancer: Prognostic and therapeutic implications. *World journal of clinical oncology*, 5(3):412.

Cluster/Subtype	Basal	Her2+	LumA	LumB	Normal
Cluster 1	155	12	0	1	3
Cluster 2	0	47	93	142	3
Cluster 3	14	9	56	20	7
Cluster 4	1	7	340	32	23

Table 1: Number of patients in each PAM50 molecular subtype and clusters identified by MIND.

**Cluster**	**Pathway Term**	**Adj. *p*-value**
**Cluster 1**	GPCR Ligand Binding	0.021
	Peptide Ligand-Binding Receptors	0.049
	Class A/1 (Rhodopsin-like Receptors)	0.092
	Potassium Channels	0.093
	Signaling By GPCR	0.102
**Cluster 2**	Bicarbonate Transporters	0.081
	Highly Calcium Permeable Postsynaptic Nicotinic Acetylcholine Receptors	0.092
	SUMOylation Of DNA Replication Proteins	0.105
	Acetylcholine Binding And Downstream Events	0.118
	Neurotransmitter Receptors And Postsynaptic Signal Transmission	0.118
**Cluster 3**	Binding And Uptake Of Ligands By Scavenger Receptors	4.6e-04
	Chylomicron Remodeling	0.001
	Cardiac Conduction	0.003
	Signaling By Retinoic Acid	0.003
	Muscle Contraction	0.003
**Cluster 4**	Estrogen-dependent Gene Expression	0.006
	ESR-mediated Signaling	0.025
	Activation Of C3 And C5	0.025
	Signaling By Nuclear Receptors	0.090
	Degradation Of Extracellular Matrix	0.090

Table 2: Top Enriched Reactome Pathways for MIND-Derived Clusters

Embedding size / Method	RF	SVM	KNN	NN	XGB
MIND_{VAE}	0.464	0.367	0.252	0.403	0.471
MIND_t	<u>0.678</u>	<u>0.651</u>	<u>0.602</u>	<u>0.591</u>	<u>0.626</u>
MIND_r	0.461	0.436	0.321	0.420	0.468
MIND	**0.709**	**0.674**	**0.675**	**0.668**	**0.659**

Table 3: Classification accuracy of different variants of MIND, CCLE. Best in **boldface**. Second best is <u>underlined</u>.

Variant/Modality	RNA	DNA methyl	CNA	miRNA	RPPA	Metabolomics
MIND_{VAE}	0.532	0.403	0.103	0.171	0.353	0.253
MIND_t	0.571	0.410	0.128	0.247	0.374	0.304
MIND_r	0.509	0.372	0.094	0.120	0.354	0.205
MIND	**0.572**	**0.447**	**0.174**	**0.244**	**0.379**	**0.338**

Table 4: Reconstruction accuracy of different variants of MIND, CCLE. Best in **boldface**. Second best is underlined.

Perplexity / Method	RF	SVM	KNN	NN	XGB
8	0.707	0.676	0.670	0.657	**0.682**
15	**0.714**	**0.678**	0.672	**0.669**	0.672
30	0.709	0.674	0.675	0.668	0.659
45	0.706	0.667	0.674	0.665	0.666
60	0.693	0.674	**0.676**	0.662	0.664

Table 5: Classification accuracy under different choices of tSNE perplexity parameter, CCLE. Best in **boldface**. Second best is underlined.

Perplexity/Modality	RNA	DNA methyl	CNA	miRNA	RPPA	Metabolomics
8	0.556	0.403	0.158	0.237	0.362	0.324
15	0.561	0.438	0.167	0.225	**0.417**	0.289
30	0.572	**0.447**	0.174	0.244	0.379	**0.338**
45	**0.574**	0.428	**0.175**	**0.275**	0.397	0.319
60	0.567	0.440	0.162	0.265	0.395	0.333

Table 6: Reconstruction accuracy under difference choices of tSNE perplexity parameter, CCLE. Best in **boldface**. Second best is underlined.

Embedding size / Method	RF	SVM	KNN	NN	XGB
8	0.684	0.658	0.665	0.657	0.655
16	0.698	0.668	0.667	0.662	0.658
32	0.702	0.676	0.670	0.667	0.671
64	**0.709**	0.674	**0.675**	**0.668**	0.659
128	0.706	**0.678**	0.661	0.663	0.669
256	0.694	0.666	0.672	0.662	**0.681**

Table 7: Classification accuracy under difference choices of embedding dimensions, CCLE. Best in **boldface**. Second best is underlined.

Embedding size / Modality	RNA	DNA methyl	CNA	miRNA	RPPA	Metabolomics
8	**0.598**	**0.478**	0.182	**0.251**	0.396	<u>0.338</u>
16	<u>0.576</u>	0.455	**0.183**	0.240	<u>0.400</u>	0.337
32	0.570	<u>0.462</u>	<u>0.181</u>	0.243	**0.409**	**0.342**
64	0.572	0.447	0.174	<u>0.244</u>	0.379	<u>0.338</u>
128	0.540	0.417	0.169	0.229	0.378	0.285
256	0.538	0.392	0.154	0.204	0.359	0.257

Table 8: Reconstruction accuracy under difference choices of embedding dimensions, CCLE. Best in **boldface**. Second best is <u>underlined</u>.

Classifier / Method	MIND	IntegrAO	JASMINE	MOVE	MSNE
RF	**0.709**	0.586	<u>0.650</u>	0.533	0.407
SVM	**0.674**	0.572	0.622	<u>0.661</u>	0.427
KNN	**0.675**	<u>0.576</u>	0.551	0.463	0.341
NN	**0.668**	0.562	0.603	<u>0.621</u>	0.363
XGB	**0.659**	0.547	<u>0.610</u>	0.497	0.367

Table 9: Cancer type classification accuracy using different classifiers, CCLE. Best in **boldface**. Second best is <u>underlined</u>.

Classifier / Method	MIND	IntegrAO	JASMINE	MOVE	MSNE
RF	**0.867**	<u>0.802</u>	0.785	0.607	0.614
SVM	**0.844**	0.748	<u>0.792</u>	0.696	0.629
KNN	**0.805**	<u>0.753</u>	0.718	0.733	0.575
NN	<u>0.837</u>	0.719	**0.841**	0.825	0.533
XGB	**0.793**	<u>0.761</u>	0.704	0.669	0.548

Table 10: Cancer type classification accuracy using different classifiers, CCMA. Best in **boldface**. Second best is <u>underlined</u>.

Classifier / Method	MIND	IntegrAO	JASMINE	MOVE	MSNE
RF	<u>0.974</u>	0.944	**0.978**	0.962	0.885
SVM	0.977	0.942	**0.981**	<u>0.980</u>	0.892
KNN	**0.976**	0.940	<u>0.972</u>	<u>0.972</u>	0.881
NN	0.980	0.951	<u>0.981</u>	**0.986**	0.904
XGB	<u>0.974</u>	0.948	**0.975**	0.964	0.891

Table 11: Cancer type classification accuracy using different classifiers, TCGA. Best in **boldface**. Second best is <u>underlined</u>.

Classifier / Method	MIND	IntegrAO	JASMINE	MOVE	MSNE
RF	**0.830**	<u>0.784</u>	0.762	0.604	0.602
SVM	**0.801**	0.735	<u>0.787</u>	0.685	0.613
KNN	**0.790**	<u>0.755</u>	0.703	0.728	0.564
NN	**0.846**	0.707	<u>0.839</u>	0.821	0.535
XGB	**0.792**	<u>0.746</u>	0.701	0.646	0.532

Table 12: Classification Accuracy of the trimmed CCMA dataset. Best in **boldface**. Second best is <u>underlined</u>. *Columns with missing data are removed.*

Dataset	Modality / Method	MIND	JASMINE	JASMINE_{aug}	MOVE
CCMA_{trim}	mRNA	**0.561**	0.069	0.111	<u>0.279</u>
	DNA methyl	**0.649**	0.202	0.455	<u>0.484</u>
	CNV	**0.858**	0.483	0.579	<u>0.681</u>

Table 13: Reconstruction Accuracy of the trimmed CCMA dataset. Pearson correlations between reconstructed and observed values of the individual modalities. JASMINE_{aug} represents the augmented JASMINE model with 64 dimensional modality-specific embeddings. For each modality, the best result is highlighted in **boldface**. Second best is <u>underlined</u>. *Columns with missing data are removed.*

Dataset / Method	MIND	IntegrAO	JASMINE	MOVE	MSNE
TCGA	2.47×10^3	<u>9.33×10^1</u>	8.47×10^3	3.35×10^3	$\mathbf{5.45 \times 10^1}$
CCLE	1.75×10^3	<u>5.72×10^1</u>	5.02×10^3	1.69×10^3	$\mathbf{3.18 \times 10^1}$
CCMA	6.13×10^2	<u>2.05×10^1</u>	1.19×10^3	6.46×10^2	$\mathbf{1.69 \times 10^1}$

Table 14: Running time (in seconds) of different methods on the three datasets. Best in **boldface**. Second best is <u>underlined</u>.

Classifier / Modality	Multi	RNA	DNA methyl	CNV	miRNA	RPPA	Metabolomics
RF	**0.709**	<u>0.678</u>	0.594	0.210	0.338	0.482	0.180
SVM	**0.674**	<u>0.645</u>	0.582	0.187	0.306	0.477	0.158
KNN	**0.675**	<u>0.674</u>	0.559	0.154	0.292	0.442	0.136
NN	**0.668**	<u>0.654</u>	0.579	0.205	0.304	0.433	0.147
XGB	**0.659**	<u>0.622</u>	0.567	0.200	0.320	0.446	0.177

Table 15: Cancer type classification accuracy using multiomics vs single modality datasets, CCLE. Best in **boldface**. Second best is <u>underlined</u>.

We thank the reviewers for their follow up comments. The following details our point-by-point response.

1 Reviewer #1

(a) The "barcode integration" proposed by the authors in this revision distinguishes itself at the problem level from "mosaic integration" by assuming that missingness is independent of batch. However, the MIND model seems to lack built-in mechanisms to remove batch effects that may exist in the data, even if they are weak or unstructured. As a result, the performance of MIND relies on the biological fidelity of the neighborhood structure in the raw data. A specific concern is that if batch effects are present in the raw data and significantly influence the neighborhood structure identified by t-SNE, this biased neighborhood information would potentially compromise the quality of the final embeddings.
(b) In cancer multi-omics databases such as TCGA, is the assumption that "modality missingness in patients is independent of batch" entirely valid? Could sample processing, sequencing centers, etc., still introduce batch effects that are weakly correlated with missingness patterns? The authors are recommended to discuss the potential risks or supplement relevant analyses regarding the two concerns above.

We thank the reviewer for these insightful follow-up comments regarding the potential influence of batch effects on the neighbourhood structure and the correlation between modality missingness and sample processing.

We acknowledge that strictly speaking the assumption of strict independence between modality missingness and batch is an idealisation. It is plausible that site-specific processing capabilities could introduce a weak correlation with modality availability. However, we emphasize that in the "barcode integration" setting, this correlation is partial and stochastic, rather than systematic: Patients in the same sequencing centre may have different modality availability patterns, while patients in different sequencing centres may share the same availability pattern. Consequently, even if there are correlations between modality availability and sequencing centres, the neighbourhood structures computed within any *single* modality are based on patients from highly heterogeneous processing origins, which naturally dilutes site-specific biases within any single affinity matrix. Indeed, the highly heterogeneous processing origins can still introduce non-independent noise to the t-SNE priors built on the affinity matrices, and potentially bias the learnt embeddings. However, our ablation studies using the Cancer Cell Line Encyclopedia (CCLE) dataset in Supplementary material provided empirical evidence showing that the t-SNE prior injects a net-positive biological signal. If the t-SNE-derived neighbourhood structures predominantly capture and propagate technical noise rather than biologically meaningful topology, we would expect the baseline model operating *without* the t-SNE informed prior (MIND_{VAE}) to match or outperform the full MIND model *with* the t-SNE informed prior. Instead, we found that the full MIND model consistently and substantially outperforms MIND_{VAE} in all classification and reconstruction tasks (Supplementary Tables S2 and S3). This demonstrates that the soft-constraint nature of the exponentially tilted prior successfully incorporates meaningful biological structure, and that the VAE reconstruction objective is capable of correcting the embeddings where the raw, noisy topological structure potentially contradicts underlying feature patterns.

In addition, MIND's architecture provides robustness to modality-specific technical artifacts.

Batch effects in multi-omics data are typically modality-specific; for example, an RNA-seq processing artifact is largely independent of a methylation array artifact. Through the patient-level aggregation step and the cross modal regularization term R in Eqn (4) of the revised text, MIND encourages consistency across modality-specific encodings, and therefore can prevent the embeddings of each modality from being dominated by technical artifacts or batch effects. This multi-modal consensus mechanism ensures that if some batch effect distorts the neighbourhood structure of one modality, the patient-level embedding obtained via mean-pooling can still remain informed by the unaffected modalities, preventing a single technical artifact from dominating the final representation. The efficacy of the cross modal regularization term R is also demonstrated in the Ablation study in Supplementary material.

We agree with the reviewer that MIND currently does not include an explicit, built-in mechanism to correct known batch covariates. Existing methods for addressing barcode missingness such as MOFA and JASMINE similarly lack native mechanisms to correct strong batch effects during training. Without appropriate preprocessing, MOFA will capture pronounced batch effects as inferred latent factors that require manual inspection, while JASMINE's reconstruction objective will encode strong technical noise directly into the final patient embeddings. Therefore, for applications where strong batch effects are present, we recommend applying established batch correction methods (e.g. ComBat (Johnson et al., 2007)) to individual modalities as a preprocessing step prior to integration. Because MIND's modular architecture accepts preprocessed modality-specific data as input, such sequential integration is seamless and consistent with established practices in bulk omics analysis. We have added the discussion above to the revised Discussion section (Pg. 8, highlighted in red) and revised Supplementary material (Pg.2, highlighted in red)

> There is an inconsistency between the subfigure labels and their corresponding descriptions in the captions of Fig. 2 and Fig. 3. The authors should carefully check these.

We have revised the captions of Fig. 2-3 in the revised main text (Pg. 21-22, highlighted in red).

> While the authors have diligently addressed each comment in the response letter, it would be beneficial for future revisions to explicitly indicate where changes have been made in the manuscript. For example, by providing specific pointers such as "Page X, Line Y" for each modification. This would greatly facilitate the review process and ensure all revisions are easily located.

We thank the reviewer for this constructive feedback. We agree that providing explicit pointers significantly facilitates the review process. As suggested, we have adopted this practice for this response. Explicit indicators such as page numbers and sections have been added to direct the reviewer to the exact locations of modifications in the revised manuscript.

2 Reviewer #2

> The authors state that comparing MIND's 64-dimensional representation to JASMINE with a higher dimensional (e.g 384) representation is advantageous for JASMINE. But the fact that MIND uses 64 dimensions does not necessarily mean that JASMINE should be used with 64 dimensions – it should be used with however many dimensions its authors recommended it to be used. If 64 is the dimension that the authors recommended for JASMINE then the current analysis is valid, but if not, making the comparison to JASMINE with 384 dimensions would show MIND's merit in a more convincing manner.

The authors thank the reviewer for pointing out this difference. We would like to clarify the motivation of our experiment design. The authors of JASMINE recommended a latent dimension of 64 in Sec 5.2 of their paper and used this setting in their TCGA analysis. We followed this setting in our experiments. We noticed that in the original paper, the embeddings were only used to perform downstream tasks. We therefore hypothesised that a latent embedding size of 64 might be ideal for supervised downstream tasks but sub-optimal for prediction and reconstruction. As a result, we added an expanded version of JASMINE, with the aim of testing whether the model can better reconstruct and predict multiomics data using modality-specific embeddings with a larger size. The results confirmed that choosing a larger embedding size for JASMINE improved its prediction performance, though the performance was still not as competitive as MIND and MOVE.

> In Tables 3 and 4 of the rebuttal, it is not clear what is MIND_t, MIND_r and MIND_{VAE}. The authors should briefly explain this, potentially in the table legends.

In Tables 3 and 4 of the rebuttal, the subscripts refer to different variants of MIND: MIND_{VAE} (without t-SNE informed prior or patient level regularization), MIND_t (with t-SNE informed prior but without patient level regularization), MIND_r (without t-SNE informed prior but with patient level regularization, $\alpha = 0.05$), and MIND (with both t-SNE informed prior and patient level regularization, $\alpha = 0.05$). We put an isotropic Gaussian prior on the embeddings whenever the t-SNE informed prior is not used. We have added the explanation to the captions of the corresponding tables in the revised Supplementary Table S2-3 (highlighted in red).

> In the analysis using CCLE comparing single-omic to multi-omic performance, the authors should compare the performance of each single-omic to the same set of patients using the multi-omic data. While we understand that the fact that more patients can be used when doing the multi-omic analysis is an advantage of MIND, using the same set of patients (each time comparing one omic to multi-omic) is a more direct comparison. The authors can include both analyses.

We updated the experiment settings in the revised text. For each modality, we now train two MIND model independently using the single modality dataset and the corresponding multimodal data of the patients present in that modality. The performance of each of the single-omic datasets are now directly compared to the same set of patients using the multi-omic data. We have updated the revised text (Pg7 highlighted in red) and Table 2. Github files have been updated accordingly.

> The authors state that they use solely cancer datasets due to data availability, but MIND would be more broadly applicable if the authors show that it can be used in multi-omic single-cell data, which are already available and are further increasing in availability (e.g. single-cell RNA + single-cell ATAC).

We agree with the reviewer that the expansion of single-cell multi-omics presents an exciting frontier for multiomics data integration. However, directly applying MIND to single-cell datasets presents fundamental methodological challenges regarding missingness structures and batch effects. We clarify the challenges and our rationale for focusing on patient-level cancer cohorts below.

As outlined in the Introduction section (Pg.2) of our revised manuscript, MIND is specifically designed to address the ”barcode integration” problem (i.e., modality availability varies across patients in a heterogeneous and nearly combinatorial manner), which structurally differs from the ”mosaic integration” typical of single-cell data (i.e., some batches of samples only contain some modalities, while other batches contain a different set of modalities). In single-cell studies, the modality availability is almost entirely tied to batch identity and sequencing technology. Because single-cell sequencing is fundamentally destructive, all cells within a given batch (e.g., a pure scRNA-seq batch or a 10x Genomics Chromium Single-Cell Multiome batch) must share identical modality availability pattern (e.g. scRNA-seq only or paired scRNA-seq+scATAC-seq), as no further measurement can be done once the batch of cells are sequenced and destroyed. State-of-the-art single-cell integration methods leverage this structured, batch-level overlap to mathematically align different batches and impute missing modalities.

However, when learning patient-level embeddings from clinical cohorts such as TCGA or CCMA, patients do not admit a natural notion of batch. Modality missingness is not structured at the batch level, but varies across patients in an arbitrary, combinatorial manner. Because the key assumption of batch-level overlap does not hold in these clinical cohorts, single-cell mosaic integration methods cannot be directly applied without substantial modification. MIND was developed to fill this specific methodological gap, positioning it alongside latent variable models such as MOVE and JASMINE rather than single-cell alignment tools.

The current iteration of MIND is optimised for the distinct missingness patterns and noise profiles of cohort-level barcode integration. In principle, MIND can be applied to single-cell multi-omics integration (e.g., scRNA-seq, scATAC-seq). However, the results are highly likely to be unreliable without substantial architectural modification. Single-cell data are known to have systematic technical noise and batch effects. Indeed, batch effects also exist in bulk omics clinical cohorts such as TCGA or CCMA, but we would like to highlight that in the “barcode integration” setting, this correlation is partial and stochastic, rather than systematic: Patients whose measurements were taken in the same sequencing centre may have different modality availability patterns, while patients in different sequencing centres may share the same availability pattern. Consequently, even if there are correlations between multiomics measurements and sequencing centres, the observed data within any *single* modality are based on patients from highly heterogeneous processing origins, which naturally dilutes site-specific biases within any single modality.

Due to strong technical noise and batch effects, analysing single-cell data robustly requires sophisticated preprocessing and alignment mechanisms, such as optimal transport, mutual nearest Neighbors (MNN) or adversarial training, to correct batch effects simultaneously with integration. MIND is proposed as a lightweight framework that incorporates a data-driven,

neighbourhood-aware prior into a multimodal VAE. Applying MIND to single-cell data without incorporating dedicated single-cell batch-correction modules would likely result in the t-SNE-derived prior capturing the strong technical batch artifacts rather than true biological manifolds.

Extending the neighbourhood-aware prior idea to single-cell architectures via optimal transport or adversarial training remains a highly promising direction for future research. We have incorporated this point in the revised Discussion section (Pg. 8, highlighted in red).

> The updated schematic figure is clearer, but it still does not show that MIND encourages the h representations for the different omics to be similar to each other, which we think is a key part of the algorithm.

We have now updated Figure 1.

> Typos

We have corrected the typos in the revised text.

3 Reviewer #3

We thank again the reviewer for supporting Early Career Researchers with peer review.

References

Johnson, W. E., Li, C., and Rabinovic, A. (2007). Adjusting batch effects in microarray expression data using empirical bayes methods. *Biostatistics*, 8(1):118–127.

We thank the reviewers for their follow up comments. The following details our point-by-point response.

1 Reviewer #1

> The authors have provided explanation regarding batch effects and the independence of modality missingness, and I am satisfied with the corresponding revisions made to the manuscript. But there is still a minor concern that the descriptions for Mosaic and Barcode integration in Figure 1 are not clearly distinguished from each other.

We thank the reviewers for their follow up comments. We have revised the caption of Fig 1 to further highlight the distinctions between Mosaic and Barcode integration.

2 Reviewer #2

We thank the reviewers for their comments and efforts.

3 Reviewer #3

We thank again the reviewer for supporting Early Career Researchers with peer review.
